# Inequalities in SARS-CoV-2 case rates by ethnicity, religion, measures of socioeconomic position, English proficiency, and self-reported disability: cohort study of 39 million people in England during the alpha and delta waves

**DOI:** 10.1136/bmjmed-2022-000187

**Published:** 2023-04-03

**Authors:** Tim Larsen, Matthew L Bosworth, Daniel Ayoubkhani, Ryan Schofield, Raghib Ali, Kamlesh Khunti, Ann Sarah Walker, Myer Glickman, Camille Harrison, Vahé Nafilyan

**Affiliations:** 1 Office for National Statistics, Newport, UK; 2 MRC Epidemiology Unit, Cambridge, UK; 3 Diabetes Research Centre, University of Leicester, Leicester, UK; 4 Nuffield Department of Medicine, Univerity of Oxford, Oxford, UK; 5 Department of Public Health Environments and Society, London School of Hygiene & Tropical Medicine, London, UK

**Keywords:** COVID-19, epidemiology, statistics, public health, socioeconomic factors

## Abstract

**Objective:**

To examine sociodemographic inequalities in people with SARS-CoV-2 during the second (alpha) and third (delta) waves of the covid-19 pandemic.

**Design:**

Retrospective, population based cohort study.

**Setting:**

Resident population of England.

**Participants:**

39 006 194 people aged 10 years and older who were enumerated in the 2011 census, registered with the NHS, and alive on 1 September 2020.

**Main outcome measures:**

Age standardised SARS-CoV-2 case rates (ie, the number of people who received a positive test result per 100 000 person weeks at risk) during the second wave (1 September 2020 to 22 May 2021) or third wave (23 May to 10 December 2021) of the pandemic. Age standardised rates were calculated by sociodemographic characteristics and adjusted rate ratios were estimated using generalised linear regression models with a Poisson distribution (models were adjusted for covariates including sex, age, geographical variables, and sociodemographic characteristics).

**Results:**

During the study period, 5 767 584 people (14.8% of the study population) tested positive for SARS-CoV-2. In the second wave, the fully adjusted relative risks of having a positive test were highest for the Bangladeshi and Pakistani ethnic groups compared with the white British group, with rate ratios of 1.75 (95% confidence interval 1.73 to 1.77) and 1.69 (1.68 to 1.70), respectively. Muslim and Sikh religious groups had fully adjusted rate ratios of 1.51 (1.50 to 1.51) and 1.64 (1.63 to 1.66), respectively, compared with the Christian group. Greater area deprivation, disadvantaged socioeconomic position, living in a care home, and low English language proficiency were also associated with higher relative risk of having a positive test. However, the inequalities among groups varied over time. Being Christian, white British, without a disability, and from a more advantaged socioeconomic position were associated with increased relative risk of testing positive during the third wave.

**Conclusion:**

Research is urgently needed to understand the large sociodemographic inequalities in SARS-CoV-2 case rates in order to inform policy interventions in future waves or pandemics.

**WHAT IS ALREADY KNOWN ON THIS TOPIC**

- People with pre-existing health conditions or disability, ethnic minority groups, elderly people, some religious groups, people with low socioeconomic status, and those living in deprived areas have been disproportionately affected by the covid-19 pandemic in terms of risk of infection and adverse outcomes

**WHAT THIS STUDY ADDS**

- Linked data on 39 million people in England were used to calculate the relative risk of testing positive for covid-19 in the community during the second and third waves of the pandemic
- During the second wave, the relative risk was highest among the Bangladeshi and Pakistani ethnic groups, the Muslim and Sikh religious groups, and people from deprived areas and of low socioeconomic status; during the third wave, being Christian, white British, without a disability, and from a more advantaged socioeconomic position were associated with increased risk of receiving a positive test
- Adjusting for geographical factors, sociodemographic characteristics, and prepandemic health status explained some, but not all, of the excess risk

**HOW THIS STUDY MIGHT AFFECT RESEARCH, PRACTICE, OR POLICY**

- Data from national, large scale testing programmes should be linked to other population level data to inform further research into the impact of covid-19 on sociodemographic groups
- These data should lead to early policy interventions targeting these groups to minimise the effect of inequalities

## Introduction

As of 18 February 2022, more than 418 million people globally have had SARS-CoV-2 infection, with more than 160 000 deaths in the UK.^1 2^ While the covid-19 pandemic has affected all areas of the UK, some groups have been disproportionally affected. Rates of covid-19 related hospital admissions and deaths have been higher among elderly people, those with pre-existing health conditions or disability,^3–5^ ethnic minority groups,^6–8^ some religious groups,^9^ people with low socioeconomic status,^10^ and those living in care homes,^11^ large households,^12^ and deprived areas.^13–15^

Less is known about sociodemographic inequalities in infection rates. Research using data from the Coronavirus Infection Survey, a large household survey representative of the UK community population, has shown that several factors were associated with SARS-CoV-2 positivity during the second wave and the early part of the third wave in the UK.^16–18^ Other studies have also highlighted non-white ethnicity, male sex, and living in an urban or more deprived area as risk factors for testing positive.^6 19 20^ However, large scale studies using national population level data sources that adjust for key confounding variables to understand the drivers of increased infection rates are limited,^21^ particularly for the third wave. Because sociodemographic inequalities in severe covid-19 outcomes appear to be largely driven by differences in infection rates, there is a clear evidence gap with which to inform national policies to reduce infection risk.

In this study, we used a large, population level dataset, comprising 2011 census data linked to administrative data sources to examine differences in SARS-CoV-2 case rates in England according to sociodemographic characteristics and disability status. We examined NHS Test and Trace data for the second and third waves of the SARS-CoV-2 pandemic, which correspond to the dominance of the alpha and delta variants, respectively. Vaccinations were also widely available during these periods of the pandemic.

## Methods

### Study data

We linked national SARS-CoV-2 positive test results obtained through pillar 1 (swab testing in UK Health Security Agency laboratories and NHS hospitals for those with a clinical need, and health and care workers) and pillar 2 (swab testing for the wider population, as set out in government guidance) to the Office for National Statistics (ONS) Public Health Data Asset (PHDA) using NHS number.

The ONS PHDA is a linked data resource combining the 2011 census, death registrations, General Practice Extraction Service Data for Pandemic Planning and Research (GDPPR)^22^ and Hospital Episode Statistics.^23^ To obtain NHS numbers, we linked the 2011 census to the 2011–13 NHS patient registers using deterministic and probabilistic matching, with an overall linkage rate of 94.6%. The NHS numbers in national testing data were incomplete, with missing values for 21% of records. To retrieve additional NHS numbers, we linked the testing data to the NHS Personal Demographics Service using deterministic matching, achieving a linkage rate of 91.4%.

**Figure 1:**
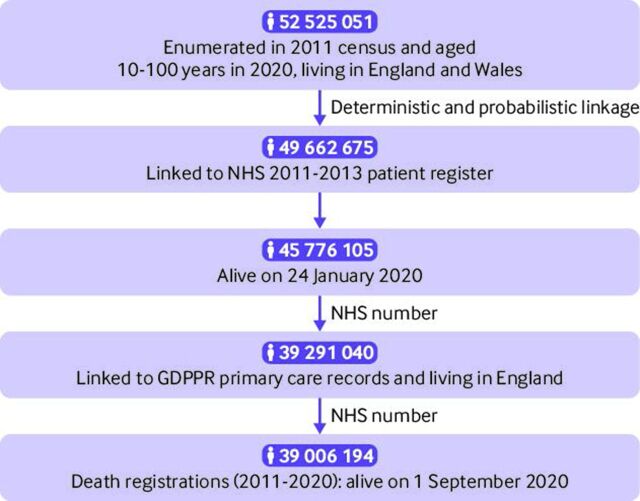
Flow diagram of how the study population was derived by combining and selecting people from different data resources. The 2011 census is linked to the patient register using deterministic and probabilistic methods with a 94.6% linkage success rate.^42 43^ GDPPR=General Practice Extraction Service Data for Pandemic Planning and Research

The study population consisted of all people aged ≥10 years living in England who were enumerated in the 2011 census, registered with a general practitioner (GP) surgery in November 2019, and alive on 1 September 2020 (figure 1). The cohort comprised 39 006 194 participants, 78.4% of the mid-year 2020 population estimate of people aged ≥10 years in England.

We used national testing data up to 10 December 2021. Out of all test results, 83.0% were linked to the ONS PHDA. We could not calculate case rates and rate ratios for the first wave because mass testing was not available.

### Characteristics and covariates

All individual level sociodemographic characteristics (sex, age, ethnic group, religious affiliation, disability status, educational attainment, National Statistics Socio-economic Classification (NS-SEC) of the household reference person, English language proficiency, country of birth) were obtained from the 2011 census. Place of residence variables (region within England and rural-urban classification^24^) and area based deprivation^25^) were derived based on postcodes held in GP records. Care home residence was retrieved from the 2019 NHS patient register. Pre-existing health conditions were derived from GDPPR data as in the QCOVID risk prediction model.^3^ We included the number of pre-existing conditions and a separate adjustment for learning disability because it could directly affect exposure to SARS-CoV-2.^26^ The number of pre-existing health conditions was included as a proxy for contact with the healthcare system, which might affect the risk of SARS-CoV-2 infection or lead to shielding. Contact with the healthcare system would also make the person more likely to be tested for SARS-CoV-2. We also adjusted for body mass index as a categorical variable with a category for missing values.

Missing data for 2011 census data were imputed using nearest neighbour donor imputation, the standard method used by the ONS to impute missing values.^27^ Because we do not have any information on which records were imputed, we could not perform multiple imputation. Therefore, the confidence intervals might not fully represent the level of uncertainty. However, the item non-response was less than 4% for all variables used in our analysis.^28^ Therefore, we would only expect this to have a minimal effect on the confidence intervals. Table S1 in supplemental file 1 lists all variables included in the analyses.

10.1136/bmjmed-2022-000187.supp1 Supplementary data

### Outcome

The outcome was receiving a positive test result (polymerase chain reaction (PCR) or lateral flow device, including positive lateral flow device tests that were not confirmed by PCR) for SARS-CoV-2. We excluded any positive tests that occurred within 120 days of an initial positive test from the same person because these might have been part of the same infection episode.^29^ We classified tests from 1 September 2020 up to and including 22 May 2021 as having occurred in the second wave of the covid-19 pandemic, with tests from 23 May 2021 to 10 December 2021 classified as being in the third wave.^17^

### Statistical analyses

We estimated age standardised SARS-CoV-2 case rates as the number of people who received a positive test result per 100 000 person weeks at risk, stratified by sociodemographic characteristics, and standardised to the 2013 European Standard Population^30^ using the approach described in the Association of Public Health Observatories’ third technical briefing.^31^ Rates were calculated separately for the second and third waves of the pandemic.

To explore differences in case rates by sociodemographic characteristics, for each factor, we compared rate ratios for testing positive for SARS-CoV-2 estimated from generalised linear regression models using a Poisson distribution, adjusted in a stepwise manner for three different sets of covariates: sex and age (model 1); sex, age, and geographical variables (region and rural-urban classification; model 2); and sex, age, geographical variables, sociodemographic characteristics (ethnicity, indices of deprivation as fifths, educational attainment, household tenure, and care home residence status), self-reported disability status, body mass index, and the number of pre-existing health conditions (model 3). Note that some of the variables in the covariate sets are considered as covariates and factor variables at different stages. Throughout the study, age is modelled using restricted natural cubic splines with 10 year age bands. The baseline rate ratios for each factor are therefore obtained under model 1, with the fully adjusted rate ratios given by model 3. This stepwise approach enables us to examine how much of the excess risk in certain groups can be accounted for by confounding factors. To account for the fact that some people died during the study period, the natural logarithm of time at risk (in days) was included in the model as an offset term.

Because of the considerable overlap between ethnicity and religion, when considering religion as our main factor of interest, we excluded ethnicity from the third covariate set. To examine the relation between ethnicity and religion in our data and their impact on rate ratios, we ran additional models using an interaction term between ethnicity and religion, adding back ethnicity to the third covariate set alongside religion as our factor. Similarly, in a separate model we investigated the interaction between ethnicity and English language proficiency (self-defined from the 2011 census), which could act as a proxy for a range of factors from cultural upbringing to the length of time a person had been in England before the 2011 census. These models are included in the online supplemental file 1.

We explored how differences in the risk of testing positive for SARS-CoV-2 changed over the course of the pandemic by fitting separate models for the second and third waves. We also fitted separate models for those aged <65 years and ≥65 years.

All analyses were conducted using R version 3.5.1 (in Cloudera Data Science Workbench) using Spark base engine 8,^32^ and the packages sparklyr^33^ and dplyr.^34^

### Patient and public involvement

We did not directly involve patients and the public in the design and conception of the study because of the pace at which this study was conducted to inform the UK government’s response to the covid-19 pandemic. The use of deidentified data precludes direct dissemination to participants. For the purpose of open access, the authors have applied a Creative Commons Attribution (CC BY) licence to any author accepted manuscript version arising. Results will also be disseminated by all coauthors through their home institutions.

## Results

Of the 39 006 194 people in our study population, 52.1% were female, the mean age was 47.6 (standard deviation 21.1) years, 81.7% identified as white British, 4.8% as white other, 2.7% as Indian, 59.5% as Christian, 25.5% as having no religious affiliation, and 5.0% as Muslim (table 1 and table S2 in online supplemental file 1). Between 1 September 2020 and 10 December 2021, 5 767 584 people (14.8% of the study population) living in England aged ≥10 years had tested positive for SARS-CoV-2; of these, 46 484 (0.8%; 0.1% of the total study population) had an infection episode in the second and third waves of the pandemic.

**Table 1:** Characteristics of the study population reported across the full study period

Variable	No (%)
**Sex**	
Male	18 697 485 (47.9)
Female	20 308 709 (52.1)
**Age group (years)**	
10-19	4 717 448 (12.1)
20-29	5 096 953 (13.1)
30-39	5 218 309 (13.4)
40-49	5 587 972 (14.3)
50-59	6 428 201 (16.5)
60-69	5 206 788 (13.4)
70-79	4 239 611 (10.9)
80-89	2 062 293 (5.3)
≥90	448 619 (1.2)
**Disability status**	
Not limited	33 694 478 (86.4)
Daily activities limited a little	3 211 382 (8.2)
Daily activities limited a lot	2 100 334 (5.4)
**Ethnic group**	
Bangladeshi	326 883 (0.8)
Black African	644 633 (1.7)
Black Caribbean	410 320 (1.1)
Chinese	203 648 (0.5)
Indian	1 055 511 (2.7)
Mixed	778 396 (2.0)
Other	993 009 (2.6)
Pakistani	854 879 (2.2)
White British	31 857 196 (81.7)
White other*	1 881 719 (4.8)
**English indices of deprivation group (fifths)**	
1 (most deprived)	7 335 236 (18.8)
2	7 620 096 (19.5)
3	7 902 220 (20.3)
4	8 040 520 (20.6)
5 (least deprived)	8 108 122 (20.8)
**Religious affiliation**	
Buddhist	155 191 (0.4)
Christian	23 191 008 (59.5)
Hindu	597 404 (1.5)
Jewish	178 494 (0.5)
Muslim	1 934 281 (5.0)
Sikh	324 447 (0.8)
No religion	9 955 732 (25.5)
Other religion	168 850 (0.4)
Not stated	2 500 787 (6.4)

*The white other group is composed of those who selected Irish, Gypsy or Irish Traveller, or other white in the 2011 census.

During the second wave, the largest differences in rates of testing positive for SARS-CoV-2 were observed for ethnicity; age standardised rates were highest in the Bangladeshi and Pakistani ethnic groups at 382.4 (95% confidence interval 377.9 to 386.9) and 373.8 (371.2 to 376.4) per 100 000 person weeks, respectively, and in the Chinese ethnic group at 90.8 (88.5 to 93.0) per 100 000 person weeks. During the third wave, however, the white British ethnic group had the highest rate at 359.7 (359.2 to 360.1) per 100 000 person weeks (table 2 and table S3 in online supplemental file 1).

There were also notable inequalities in case rates by religious affiliation. During the second wave of the pandemic, rates per 100 000 person weeks were highest for people who identified as Muslim (334.9, 333.3 to 336.5) or Sikh (321.6, 318.3 to 325.0). Rates were lowest for people in the other religion group (142.9, 139.4 to 146.3) and the Buddhist group (143.3, 139.9 to 146.7). During the third wave, those who identified as Christian had the highest rates at 353.8 (353.3 to 354.3) per 100 000 person weeks, whereas the lowest rates were found in the Buddhist and Muslim groups at 221.4 (216.3 to 226.4) and 226.7 (225.4 to 228.1) per 100 000 person weeks, respectively.

**Table 2:** Age standardised SARS-CoV-2 case rates (per 100 000 person weeks) by sociodemographic characteristics and wave of the pandemic

Characteristic	Wave two (1 September 2020 to 22 May 2021)		Wave three (23 May to 10 December 2021)	
	No of cases	Rate (95% CI)	No of cases	Rate (95% CI)
**Sex**				
Female	1 357 898	189.1 (188.8 to 189.4)	1 796 143	347.8 (347.3 to 348.3)
Male	1 090 708	162.7 (162.4 to 163.0)	1 569 319	316.3 (315.8 to 316.8)
**Disability status**				
No disability—not limited	2 147 056	174.0 (173.8 to 174.2)	3 134 229	337.6 (337.3 to 338.0)
With disability—limited a little	173 719	162.9 (161.9 to 163.9)	146 457	272.0 (270.1 to 273.9)
With disability—limited a lot	127 831	159.9 (158.7 to 161.1)	84 776	212.6 (210.6 to 214.6)
**Ethnic group**				
Bangladeshi	43 449	382.4 (377.9 to 386.9)	23 756	229.9 (226.3 to 233.5)
Black African	47 855	200.2 (198.0. to 202.4)	41 958	198.4 (196.2 to 200.5)
Black Caribbean	27 748	184.6 (182.3 to 186.8)	28 941	266.4 (263.2 to 269.5)
Chinese	6811	90.8 (88.5 to 93.0)	9031	162.5 (159.0 to 165.9)
Indian	102 001	267.3 (265.6 to 269.0)	80 550	265.9 (264.0 to 267.8)
Mixed	55 724	183.5 (181.5 to 185.5)	88 670	303.5 (301.0 to 305.9)
Other	87 798	238.0 (236.3 to 239.7)	68 648	225.5 (223.7 to 227.3)
Pakistani	110 638	373.8 (371.2 to 376.4)	62 132	233.1 (231.0 to 235.2)
White British	1 851 398	165.3 (165.0 to 165.5)	2 824 792	359.7 (359.2 to 360.1)
White other	115 184	166.7 (165.7 to 167.8)	136 984	260.3 (258.8 to 261.8)
**Education level**				
No qualification	356 433	150.4 (149.8 to 151.0)	258 959	175.6 (174.8 to 176.4)
Apprenticeship	58 991	138.0 (136.7 to 139.3)	64 967	224.2 (222.1 to 226.2)
Level 1	300 887	144.3 (143.7 to 144.9)	335 274	211.2 (210.5 to 211.9)
Level 2	347 997	142.4 (141.9 to 142.9)	410 814	219.1 (218.4 to 219.8)
Level 3	270 548	137.7 (137.2 to 138.3)	343 028	223.3 (222.5 to 224.1)
Level 4	456 144	119.3 (118.6 to 119.9)	656 680	212.4 (211.4 to 213.5)
Other	108 109	156.4 (155.4 to 157.5)	83 920	165.7 (164.3 to 167.1)
**English indices of deprivation group (fifths)**				
1 (most deprived)	581 068	218.7 (218.1 to 219.2)	644 804	311.8 (311.0 to 312.6)
2	527 010	191.2 (190.7 to 191.7)	649 484	317.3 (316.6 to 318.1)
3	472 664	168.5 (168.0 to 169.0)	666 824	331.5 (330.7 to 332.3)
4	450 659	160.3 (159.8 to 160.8)	690 861	347.5 (346.7 to 348.3)
5 (least deprived)	417 205	148.0 (147.6 to 148.5)	713 489	358.1 (357.3 to 359.0)
**Religious affiliation**				
Buddhist	8043	143.3 (139.9 to 146.7)	8860	221.4 (216.3 to 226.4)
Christian	1 406 889	177.3 (177.0 to 177.6)	1 920 206	353.8 (353.3 to 354.3)
Hindu	49 248	227.3 (225.2 to 229.4)	45 158	265.2 (262.7 to 267.7)
Jewish	11 730	189.8 (186.3 to 193.3)	13 298	293.1 (288.0 to 298.1)
Muslim	228 476	334.9 (333.3 to 336.5)	139 064	226.7 (225.4 to 228.1)
Sikh	37 471	321.6 (318.3 to 325.0)	26 322	286.0 (282.5 to 289.5)
No religion	564 183	147.2 (146.8 to 147.6)	1 000 330	336.2 (335.6 to 336.9)
Other religion	8284	142.9 (139.4 to 146.3)	10 725	267.6 (261.6 to 273.7)
Not stated	134 282	151.9 (151.1 to 152.7)	201 499	304.9 (303.5 to 306.2)

In the second wave, the Bangladeshi ethnic group had the highest rate ratio of testing positive for SARS-CoV-2 relative to the white British ethnic group (table 3, with a full list of model results in table S4 in online supplemental file 1); adjusting for age and sex only, the rate ratio was 2.03 (95% CI 2.01 to 2.05), whereas the model 3 rate ratio was 1.75 (1.73 to 1.77). Geography, sociodemographic factors, and prepandemic health status accounted for 27.2% of the increased relative risk of testing positive for SARS-CoV-2 among the Bangladeshi ethnic group during the second wave of the pandemic. During the third wave, however, the relative risk of testing positive for SARS-CoV-2 was lower for all ethnic minority groups compared with the white British group, including the white other group.

**Table 3:** Adjusted rate ratios (95% confidence intervals) of receiving a positive test for SARS-CoV-2 by sociodemographic characteristics and wave of the pandemic

Characteristic	Wave two (1 September 2020 to 22 May 2021)			Wave three (23 May to 10 December 2021)		
	Model 1	Model 2	Model 3	Model 1	Model 2	Model 3
**Disability status**						
Not limited	1 (reference)	1 (reference)	1 (reference)	1 (reference)	1 (reference)	1 (reference)
Limited a little	1.03 (1.02 to 1.03)	1.01 (1.00 to 1.01)	0.92 (0.92 to 0.93)	0.85 (0.85 to 0.86)	0.85 (0.84 to 0.85)	0.87 (0.86 to 0.87)
Limited a lot	1.15 (1.15 to 1.16)	1.10 (1.10 to 1.11)	0.94 (0.93 to 0.94)	0.74 (0.73 to 0.74)	0.73 (0.72 to 0.73)	0.77 (0.77 to 0.78)
**Ethnic group**						
White British	1 (reference)	1 (reference)	1 (reference)	1 (reference)	1 (reference)	1 (reference)
Bangladeshi	2.03 (2.01 to 2.05)	1.83 (1.81 to 1.84)	1.75 (1.73 to 1.77)	0.59 (0.58 to 0.60)	0.65 (0.64 to 0.66)	0.68 (0.67 to 0.68)
Black African	1.15 (1.14 to 1.16)	1.05 (1.04 to 1.06)	1.05 (1.04 to 1.06)	0.55 (0.54 to 0.55)	0.61 (0.61 to 0.62)	0.64 (0.64 to 0.65)
Black Caribbean	1.11 (1.10 to 1.12)	1.01 (1.00 to 1.02)	0.97 (0.96 to 0.98)	0.78 (0.77 to 0.79)	0.88 (0.87 to 0.89)	0.91 (0.89 to 0.92)
Chinese	0.54 (0.53 to 0.56)	0.51 (0.50 to 0.52)	0.55 (0.54 to 0.57)	0.45 (0.44 to 0.46)	0.47 (0.46 to 0.48)	0.49 (0.48 to 0.50)
Indian	1.59 (1.58 to 1.60)	1.46 (1.45 to 1.47)	1.50 (1.49 to 1.51)	0.75 (0.74 to 0.75)	0.80 (0.79 to 0.80)	0.79 (0.78 to 0.80)
Mixed	1.10 (1.09 to 1.11)	1.04 (1.03 to 1.05)	1.04 (1.04 to 1.05)	0.85 (0.85 to 0.86)	0.90 (0.90 to 0.91)	0.92 (0.92 to 0.93)
Other	1.41 (1.40 to 1.42)	1.30 (1.29 to 1.31)	1.31 (1.30 to 1.32)	0.63 (0.62 to 0.63)	0.69 (0.68 to 0.69)	0.72 (0.71 to 0.72)
Pakistani	2.01 (2.00 to 2.02)	1.76 (1.75 to 1.77)	1.69 (1.68 to 1.70)	0.60 (0.60 to 0.61)	0.61 (0.60 to 0.61)	0.62 (0.61 to 0.62)
White other	1.00 (1.00 to 1.01)	0.97 (0.96 to 0.98)	1.00 (1.00 to 1.01)	0.73 (0.73 to 0.74)	0.79 (0.79 to 0.80)	0.83 (0.82 to 0.83)
**Education level**						
No qualification	1 (reference)	1 (reference)	1 (reference)	1 (reference)	1 (reference)	1 (reference)
Apprenticeship	0.93 (0.92 to 0.94)	0.98 (0.97 to 0.99)	1.05 (1.04 to 1.06)	1.29 (1.28 to 1.30)	1.26 (1.25 to 1.27)	1.17 (1.16 to 1.18)
Level 1	0.91 (0.91 to 0.92)	0.95 (0.94 to 0.95)	0.99 (0.99 to 1.00)	1.15 (1.15 to 1.16)	1.15 (1.15 to 1.16)	1.10 (1.09 to 1.10)
Level 2	0.90 (0.90 to 0.90)	0.94 (0.94 to 0.95)	1.00 (1.00 to 1.01)	1.19 (1.18 to 1.20)	1.19 (1.18 to 1.19)	1.11 (1.11 to 1.12)
Level 3	0.88 (0.87 to 0.88)	0.92 (0.92 to 0.92)	0.99 (0.98 to 0.99)	1.22 (1.21 to 1.22)	1.22 (1.21 to 1.22)	1.14 (1.13 to 1.14)
Level 4	0.72 (0.72 to 0.73)	0.76 (0.75 to 0.76)	0.82 (0.82 to 0.82)	1.17 (1.17 to 1.18)	1.21 (1.20 to 1.21)	1.14 (1.14 to 1.15)
Other	1.02 (1.01 to 1.03)	1.02 (1.01 to 1.02)	1.02 (1.01 to 1.02)	0.93 (0.92 to 0.93)	0.98 (0.97 to 0.99)	1.06 (1.05 to 1.06)
**English indices of deprivation group (fifths)**						
1 (most deprived)	1.45 (1.45 to 1.46)	1.27 (1.27 to 1.28)	1.17 (1.16 to 1.17)	0.88 (0.88 to 0.88)	0.84 (0.83 to 0.84)	0.93 (0.93 to 0.93)
2	1.29 (1.28 to 1.29)	1.21 (1.20 to 1.21)	1.14 (1.13 to 1.14)	0.90 (0.89 to 0.90)	0.91 (0.91 to 0.91)	0.96 (0.96 to 0.97)
3	1.14 (1.13 to 1.14)	1.13 (1.13 to 1.14)	1.10 (1.09 to 1.10)	0.93 (0.93 to 0.93)	0.95 (0.94 to 0.95)	0.98 (0.97 to 0.98)
4	1.08 (1.08 to 1.09)	1.09 (1.08 to 1.09)	1.07 (1.06 to 1.07)	0.97 (0.97 to 0.98)	0.97 (0.97 to 0.98)	0.99 (0.98 to 0.99)
5 (least deprived)	1 (reference)	1 (reference)	1 (reference)	1 (reference)	1 (reference)	1 (reference)
**Religious affiliation**						
Christian	1 (reference)	1 (reference)	1 (reference)	1 (reference)	1 (reference)	1 (reference)
Buddhist	0.81 (0.79 to 0.82)	0.80 (0.78 to 0.82)	0.84 (0.82 to 0.86)	0.63 (0.62 to 0.65)	0.68 (0.66 to 0.69)	0.71 (0.69 to 0.72)
Hindu	1.27 (1.26 to 1.29)	1.20 (1.19 to 1.21)	1.24 (1.23 to 1.26)	0.76 (0.75 to 0.77)	0.85 (0.84 to 0.86)	0.85 (0.84 to 0.86)
Jewish	1.07 (1.05 to 1.09)	0.99 (0.98 to 1.01)	1.04 (1.02 to 1.06)	0.84 (0.83 to 0.86)	0.97 (0.95 to 0.98)	0.95 (0.93 to 0.96)
Muslim	1.71 (1.71 to 1.72)	1.55 (1.55 to 1.56)	1.51 (1.50 to 1.51)	0.60 (0.60 to 0.61)	0.64 (0.64 to 0.65)	0.67 (0.67 to 0.67)
Sikh	1.76 (1.75 to 1.78)	1.65 (1.63 to 1.66)	1.64 (1.63 to 1.66)	0.81 (0.80 to 0.82)	0.86 (0.85 to 0.87)	0.85 (0.84 to 0.86)
No religion	0.85 (0.85 to 0.85)	0.87 (0.87 to 0.87)	0.88 (0.87 to 0.88)	0.96 (0.95 to 0.96)	0.96 (0.95 to 0.96)	0.97 (0.97 to 0.97)
Other religion	0.79 (0.77 to 0.80)	0.80 (0.79 to 0.82)	0.81 (0.79 to 0.83)	0.77 (0.76 to 0.79)	0.80 (0.78 to 0.81)	0.81 (0.79 to 0.82)
Not stated	0.87 (0.87 to 0.88)	0.88 (0.88 to 0.89)	0.89 (0.88 to 0.89)	0.87 (0.87 to 0.88)	0.88 (0.88 to 0.89)	0.89 (0.89 to 0.90)

Model 1, adjusted for age and sex only; model 2, adjusted for age, sex, and geographical variables (region and rural-urban classification); model 3, adjusted for age, sex, geographical variables, sociodemographic characteristics (ethnicity, indices of deprivation as fifths, educational attainment, household tenure, and care home residence status), self-reported disability status, body mass index, and the number of pre-existing health conditions. Note that for religion the fully adjusted model (model 3) does not adjust for ethnicity.

In the second wave, for religious affiliation, the highest rate ratio of testing positive for SARS-CoV-2 (compared with the Christian group) was observed for people identifying as Sikh; when adjusting for age and sex, the rate ratio was 1.76 (95% confidence interval 1.75 to 1.78), reducing to 1.64 (1.63 to 1.66) in model 3. This suggests that geography, sociodemographic factors (not including ethnicity) and prepandemic health status only explained 15.8% of the increased excess risk of testing positive for SARS-CoV-2 among people identifying as Sikh during the second wave of the pandemic. During the third wave, the relative risk of testing positive for SARS-CoV-2 was highest among those identifying as Christian; the lowest rate ratio was observed in the Muslim population at 0.67 (0.67 to 0.67), while the highest was for the no religion group at 0.97 (0.97 to 0.97).

We found large differences and variations in risk over time according to care home residency status. In the second wave, the model 3 rate ratio of testing positive for people living in a care home was 4.30 (4.25 to 4.35) compared with those not in a care home, whereas in the third wave the model 3 rate ratio was 1.32 (1.28 to 1.36).

Several other factors were independently associated with SARS-CoV-2 infection. For example, people living in urban areas had higher relative risk of testing positive for SARS-CoV-2 than those living in rural areas during the second and third waves. Living in a more deprived area was also associated with higher relative risk of testing positive during the second wave (rate ratio for most deprived group 1.45, 95% confidence interval 1.45 to 1.46 compared with the least deprived group) but not in the third wave (least deprived group 0.88, 0.88 to 0.88). During the second wave, people who reported that English was not their main language had higher relative risk of testing positive for SARS-CoV-2 than those who reported speaking English as their main language after adjusting for other factors (rate ratio for those who do not speak English well or at all 1.48, 95% confidence interval 1.47 to 1.49 when adjusting for age and sex; 1.10, 1.09 to 1.11 in model 3). Conversely, during the third wave, the relative risk of testing positive among people who did not speak English as their main language was lower than those whose main language was English (rate ratio for those who do not speak English well or at all 0.83, 0.82 to 0.84 in model 3).

People with a disability who were limited a lot in their daily activities had increased relative risk of testing positive during the second wave after adjusting for age and sex only (rate ratio for those limited a lot 1.15, 95% confidence interval 1.15 to 1.16), but had lower relative risk than people without a disability in model 3 (rate ratio for those limited a lot 0.94, 0.93 to 9.94). In the third wave, people with a disability had lower relative risk of testing positive than those without a disability across all models. Odds ratios are shown as plots S1-S3 in the online supplemental file 1.

As an exploratory analysis, we stratified the data by broad age group (<65 years *v* ≥65 years). Among people aged <65 years (table S5 in online supplemental file 1), all ethnic minority groups had lower relative risk of testing positive than the white British group during the third wave, as was observed in the main models. Conversely, during the third wave among people aged ≥65 years (table S6 in online supplemental file 1), the relative risk of testing positive from model 3 was highest for the Bangladeshi ethnic group (rate ratio 1.61, 95% confidence interval 1.50 to 1.72).

We also performed a sensitivity analysis for missing body mass index data by running a model after filtering out all those with missing data (classified as unknown; see table S1 in online supplemental file 1). The results after this filtering give similar model coefficients, which are provided in online supplemental file 2 and online supplemental file 3. Results of the models with interactions are included in online supplemental file 4 and online supplemental file 5).

10.1136/bmjmed-2022-000187.supp2 Supplementary data

10.1136/bmjmed-2022-000187.supp3 Supplementary data

10.1136/bmjmed-2022-000187.supp4 Supplementary data

10.1136/bmjmed-2022-000187.supp5 Supplementary data

## Discussion

### Main findings

Our analysis using population level linked data in England shows that there were major inequalities in covid-19 case rates in people aged ≥10 years during the second and third waves for several sociodemographic characteristics, most notably by ethnic group, religious affiliation, and rural-urban classification. During the second wave, case rates were highest among Bangladeshi and Pakistani ethnic groups, with adjustments for geographical variables, socioeconomic factors, and pre-existing health conditions accounting for 27.2% and 31.7% of the excess risk, respectively. For religious affiliation, those who identified as Muslim or Sikh had the highest rates, with adjustments only accounting for 27.2% and 15.8% of the excess risk, respectively. While some differences were found by deprivation and other sociodemographic factors, these were less pronounced than for ethnicity or religious affiliation. However, there is considerable overlap between ethnicity and religion; 93.4% of people from the Pakistani and Bangladeshi ethnic groups within the study self-identified as Muslim. The highest rates were seen among people from the most deprived areas, even in model 3. Those who do not speak English well or at all were at greater risk of having a positive test than those with English as their main language, with adjustments for geographical variables, socioeconomic factors, and pre-existing health conditions accounting for 79.2% of the excess risk.

For the third wave, corresponding to the emergence of the delta variant, we observed a different pattern for several factors. The white British ethnic group had the highest case rates and rate ratios, while those who self-identified as Christian had the highest rates among religious affiliations. Case rates also became highest among people born in the UK and whose main language was English. A potential reason is that levels of population immunity were higher for the groups that had the highest case rates in the first and second waves, even considering the potential for reinfection.^35^

Changes in the rate ratios observed in wave three compared with wave two could also be due to changes in testing behaviours in response to rollout of vaccination, changes in the perceived risk of infection or reinfection, and policy changes related to isolation periods and compensation after testing positive for SARS-CoV-2. Rates of access to sick pay in England and Wales were lower among South Asian workers than white British workers^36^ and it was more difficult for ethnic minority groups to access Test and Trace services,^37^ which probably had an impact on case rates among these groups. Interestingly, when stratifying these models by broad age groups (<65 years *v* ≥65 years) as an exploratory analysis, we found that the rate ratios for all ethnic minority groups were higher in the model restricted to people aged ≥65 years compared with the unrestricted model and the model restricted to those aged <65 years. These results could indicate the presence of further factors affecting the underlying risk of infection and the likelihood of being tested, such as living in multigenerational and overcrowded households. This finding is consistent with the continued increased risk of mortality during the third wave for ethnic minority groups compared with the white British population.^7 38^

### Comparison with other studies

Our findings are consistent with results from the Coronavirus Infection Survey, which found that between September 2020 and May 2021, people living in urban areas and deprived areas, and of a younger age were most likely to test positive in the UK.^16^ Studies using UK covid-19 surveillance data have also suggested that black and South Asian ethnic groups were more likely to test positive than white British people in England.^6 39^ In addition, our results support previous analyses using UK administrative data that have shown higher age standardised case rates among ethnic minority groups until June 2021, when rates increased among the white population.^38^ Similar patterns of increased infection in the most deprived areas and among minority ethnic groups have been observed worldwide.^10 40^

Studies have shown that covid-19 vaccinations significantly reduced the risk of SARS-CoV-2 infection.^18^ From December 2020 onwards, unadjusted vaccination uptake rates were lower among adults from ethnic minority groups, people living in the most deprived areas, those self-reporting as having a disability, people younger in age, those who did not speak English as their first language, and people who belonged to a lower socioeconomic group.^38 41^ These data are consistent with our findings when adjusting for age and sex only during the second wave, suggesting that lower vaccine uptake rates for certain groups and younger people might contribute to case rate inequalities. Although vaccination rates were lower for the Bangladeshi and Pakistani groups than the white British population, the lowest rates were found in black African and black Caribbean groups.

### Strengths and limitations

The primary strength of the study is using nationwide linked population level data that combine a diverse set of demographic and socioeconomic factors from the 2011 census with timely data on national SARS-CoV-2 testing. Unlike studies based solely on electronic health records, our study is based on self-identified ethnicity, limiting the potential for factor misclassification bias. We also have information on a wide range of sociodemographic factors not typically available in electronic health records, such as religion, main language, and educational attainment. Another strength is the size of the dataset, comprising 78.4% of people aged ≥10 years living in England in 2020. Therefore, this study is sufficiently powered to detect small differences in the relative risk of testing positive for SARS-CoV-2 by detailed characteristics after adjusting for confounding factors and interactions with age.

An important limitation is that the PHDA only contains information on people who were enumerated in the 2011 census. Therefore, it excludes people living in England in 2011 who did not participate in the 2011 census (estimated to be approximately 5% of the population at the time); respondents who could not be linked to the 2011-13 NHS patient registers (5.4% of census respondents); people who have immigrated since 2011; children <10 years old in 2021; and people not registered with a GP surgery or who had opted out of GDPPR. Additionally, the NHS patient register is known to have coverage issues,^42^ with undercoverage of specific groups such as migrants and recent returnees to the UK, armed forces and dependants, prisoners, and people registered only with private practices. Therefore, because our study population is based on the PHDA, specific groups might not be adequately covered,^43^ which could result in biased estimates of relative risks for some groups. However, the coverage is high and the biases are probably small.

A further limitation is that many of the sociodemographic variables were derived from the 2011 census. Some of these characteristics (for example, disability status, English language proficiency, and NS-SEC) might have changed since the 2011 census and might not accurately reflect peoples’ circumstances during the pandemic. Some unaccounted factors might also exists that could contribute to the inequalities in case rates observed across ethnicities, such as current occupation or household size, with Pakistani and Bangladeshi groups being most likely to work in occupations which carry greater risk of infection^37^ and live in overcrowded households with poor ventilation.^44 45^ Because our occupation data are from the 2011 census, we have used the NS-SEC of the household reference person to give wider coverage of age groups. Using this as a proxy for occupation means people could have changed NS-SEC categories since the 2011 census, particularly those who are not the household reference person and have moved out.

National SARS-CoV-2 testing data do not provide a representative measure of infections because people are more likely to get a test for covid-19 if they have symptoms, as they are advised to do, and because there might also be other biases in the choice to get a test. About 40% of people who tested positive in the Coronavirus Infection Survey did not develop symptoms within 35 days of testing positive.^29^ Therefore, these figures are likely to under-represent the number of people without symptoms and so might not be generalisable to all infections in the population. Additionally, people in certain occupations and school children are required to undergo regular testing, and so might be more likely to test positive for covid-19 as a result of higher testing rates. Adherence to testing has been shown to be lower among men and boys, those of younger age, and people of lower socioeconomic status,^46^ meaning inequalities in case rates are likely to be underestimated.

We were not able to account for the impact of lockdown measures on relative risks because these varied over time throughout the waves and differed by geographical areas. These policies were also not consistent across occupations and so varying rates of sociodemographic characteristics across regions and occupations could lead to differential risks which are not accounted for in this study.

Different diagnostic tests have been used for identifying SARS-CoV-2 infection, with the gold standard being reverse transcription PCR testing, a technique based on amplifying genetic material present in a sample to confirm the presence of the virus. All test types have been found to have high specificity, meaning that false positives are rare, while the test sensitivities have been found to differ across type of tests.^47^ With the accuracy of tests being affected by the timing and the conditions of the test, and in people with symptoms the ability and willingness to identify their symptoms and seek a test,^48 49^ the case rates reported in this study are probably underestimates. A large scale population study would be valuable to understand the differences in test seeking behaviours and estimate the probability of being tested for SARS-CoV-2 according to sociodemographics.

### Conclusion

SARS-CoV-2 case rates were found to vary considerably across different sociodemographic groups, particularly ethnicity and religion, in the second and third waves of the covid-19 pandemic. Further research is needed to understand why these inequalities exist and how they can best be addressed through policy interventions. Continued surveillance is essential to ensure that changes in the patterns of infection are identified early to inform public health interventions.

## Data Availability

No data are available.
