# Supplementary material for: Inequalities in SARS-CoV-2 case rates by ethnicity, religion, measures of socioeconomic position, English proficiency, and self-reported disability: cohort study of 39 million people in England during the alpha and delta waves
Source: BMJ Med. 2023 Apr 3;2(1):e000187. doi: 10.1136/bmjmed-2022-000187 (PMC10568121; doi:10.1136/bmjmed-2022-000187)
Supplement: Supplementary data [file bmjmed-2022-000187supp001.pdf]

Supplementary material

Table S1: Sources of variables used in the analyses

Variable	Coding	Source
**Positive test for SARS-CoV-2**	Tested positive for SARS-CoV-2 between 1 September 2020 and 10 December 2021 and result recorded in national testing data	NPEx/SGSS
**Age**	Restricted natural cubic spline (using 10-year age bands)	2011 Census
**Sex**	Female, male	2011 Census
**Ethnicity**	White British, Bangladeshi, Black African, Black Caribbean, Chinese, Indian, Mixed, Other, Pakistani, White Other	2011 Census
**Religious affiliation**	Christian, Buddhist, Hindu, Jewish, Muslim, Sikh, no religion, other religion, religion not stated	2011 Census
**Region**	North East, North West, Yorkshire and The Humber, East Midlands, West Midlands, East of England, London, South East, South West	GDPPR
**Rural-Urban Classification**	Major or minor conurbation; city and town; town and Fringe; villages, hamlets and isolated dwellings	GDPPR
**English Indices of Deprivation**	Dummy variables representing quintile groups of deprivation, with 1 as the most deprived and 5 as the least deprived groups.	GDPPR
**National Statistics Socio-economic classification (NS-SEC)**	Higher managerial, administrative and professional occupations;<br>Lower managerial, administrative and professional occupations;<br>Intermediate occupations;<br>Small employers and own account workers;	2011 Census

	Lower supervisory and technical occupations;<br>Semi-routine occupations;<br>Routine occupations;<br>Never worked and long-term unemployed;<br>Not in a household;<br>Not classified	
**Residence type**	Dummy variables representing private household or other communal establishment, or care home residency	GDPPR/2011 Census
**Household tenure**	Own, social rented, private rented, other	2011 Census
**Country of birth**	UK, non-UK	2011 Census
**English language proficiency**	Main language, other	2011 Census
**Level of highest qualification**	Degree, A-level or equivalent, GCSE or equivalent, no qualification, other	2011 Census
**Disability**	Non-disabled, disabled (daily activities limited a little), disabled (daily activities limited a lot)	2011 Census
**Body Mass Index (kg/m2)**	< 18.5, 18.5 to <25, 25 to <30, >= 30, unknown	GDPPR
**Learning disability**	No learning disability, Down's syndrome, other learning disability	GDPPR
**Pre-existing conditions**	Number of pre-existing conditions	GDPPR/HES

NPEx, National Pathology Exchange; SGSS, Second Generation Surveillance System; GCSE, General Certificate of Secondary Education;GDPPR, General Practice Extraction Service Data for Pandemic Planning and Research; HES, Hospital Episode Statistics

Table S2: Characteristics of the study population reported across the full study period (variables not in the main text tables).

Variable	Level	Count (%)
Education level	No qualification	6,039,757 (15.5)
	Apprenticeship	1,131,625 (2.9)
	Level 1	4,557,085 (11.7)
	Level 2	5,174,885 (13.3)
	Level 3	4,065,049 (10.4)
	Level 4	8,927,314 (22.9)
	Not classified	7,482,144 (19.2)
	Other	1,628,335 (4.2)
Household tenure	Private rented	5,732,235 (14.7)
	Social rented	5,953,221 (15.3)
	Owned	26,395,380 (67.7)
	Other (e.g., live rent free)	619,926 (1.6)
Care home status	No	38,823,660 (99.5)
	Yes	182,534 (0.5)
National Statistics Socio-Economic Classification of the household reference person	1 Higher managerial, administrative and professional occupations	5,541,223 (14.2)
	2 Lower managerial, administrative and professional occupations	9,072,504 (23.3)
	3 Intermediate occupations	4,024,100 (10.3)
	4 Small employers and own account workers	5,145,160 (13.2)
	5 Lower supervisory and technical occupations	3,407,288 (8.7)
	6 Semi-routine occupations	4,925,771 (12.6)
	7 Routine occupations	4,571,754 (11.7)
	8 Never worked and long-term unemployed	1,437,647 (3.7)
	Not in a household	305,432 (0.8)
	Not classified	575,315 (1.5)
Country of birth	UK	34,244,696 (87.8)

	Non-UK	4,761,498 (12.2)
**English language proficiency**	Main language	36,311,243 (93.1)
	Well or very well	2,114,632 (5.4)
	Not well or not at all	580,319 (1.5)
**Rural Urban Classification**	Major or minor conurbation	14,546,114 (37.3)
	City and town	17,317,517 (44.4)
	Town and fringe	3,561,427 (9.1)
	Villages, hamlets and isolated dwellings	3,581,136 (9.2)
**BMI category**	Underweight	366,743 (0.9)
	Ideal	6,300,076 (16.2)
	Overweight	6,386,147 (16.4)
	Obese	5,512,880 (14.1)
	Missing	20,440,348 (52.4)
**Learning condition**	Does not have a learning condition	38,574,896 (98.9)
	Has a learning condition	431,298 (1.1)

Table S3: Age-standardised SARS-CoV-2 case rates (per 100,000 person-weeks) by socio-demographic characteristics and wave of the pandemic (variables not in the main text tables).

Exposure		Wave two (1 September 2020 to 22 May 2021) Number of cases	Rate	Lower CI	Upper CI	Wave three (23 May 2021 onwards) Number of cases	Rate	Lower CI	Upper CI
Household tenure	Private rented	367,298	168.4	167.8	169.1	548,583	299.4	298.5	300.3
	Social rented	419,093	193.1	192.5	193.7	508,011	296.3	295.5	297.1
	Owned	1,601,009	177.8	177.5	178.1	2,237,339	355.1	354.6	355.6
	Other	38,571	169.3	167.6	171.0	50,985	300.6	298.0	303.3
National Statistics Socio-Economic Classification of the household reference person	Higher managerial, administrative and professional occupations	139,872	102.0	100.7	103.3	229,509	211.3	208.8	213.7
	Lower managerial, administrative and professional occupations	369,283	128.3	127.7	128.9	498,925	228.8	227.7	229.9
	Intermediate occupations	250,852	141.0	140.3	141.6	299,393	228.8	227.8	229.8
	Small employers and own account workers	153,985	127.6	126.7	128.6	163,628	189.7	188.1	191.3
	Lower supervisory and technical occupations	134,166	142.5	141.7	143.3	144,680	213.1	211.8	214.3
	Semi-routine occupations	299,012	158.0	157.4	158.6	293,050	211.9	211.1	212.7
	Routine occupations	208,805	145.7	145.1	146.4	203,723	202.9	202.0	203.9
	Never worked and long-term unemployed	110,573	153.8	152.9	154.8	92,397	158.8	157.8	159.8
Country of birth	UK	2,089,141	171.7	171.5	171.9	3,081,174	345.0	344.6	345.4
	Non-UK	359,465	203.1	202.4	203.9	284,288	238.2	237.1	239.4
English language proficiency	Main language	2,211,286	171.6	171.4	171.8	3,191,368	342.2	341.9	342.6
	Speak English very well or well	187,607	239.4	238.2	240.6	140,685	228.2	226.8	229.5

Exposure		Wave two (1 September 2020 to 22 May 2021)				Wave three (23 May 2021 onwards)			
		Number of cases	Rate	Lower CI	Upper CI	Number of cases	Rate	Lower CI	Upper CI
	Do not speak English well or at all	49,713	238.8	236.2	241.5	33,409	194.4	191.6	197.2
Rural-Urban Classification	Cities and towns	1,005,795	162.3	162.0	162.6	1,565,237	350.0	349.4	350.5
	Major or minor conurbations	1,129,050	215.0	214.6	215.3	1,223,891	308.7	308.1	309.2
	Towns and fringes	171,629	139.9	139.2	140.5	307,205	360.1	358.8	361.4
	Villages, hamlets and isolated dwellings	142,132	118.8	118.1	119.4	269,129	332.3	331.0	333.7
Region	North East	132,204	194.7	193.6	195.7	204,171	420.4	418.6	422.2
	North West	400,613	218.2	217.5	218.8	487,936	363.6	362.5	364.6
	Yorkshire and the Humber	270,098	192.0	191.3	192.7	384,690	375.8	374.6	377.0
	East Midlands	218,633	177.9	177.1	178.6	320,396	363.1	361.8	364.4
	West Midlands	282,221	193.6	192.9	194.3	354,840	332.9	331.8	334.0
	East of England	250,486	158.4	157.8	159.1	352,650	308.6	307.6	309.7
	London	406,794	205.4	204.7	206.0	371,473	241.6	240.8	242.4
	South East	343,314	150.6	150.1	151.1	525,119	319.4	318.6	320.3
	South West	144,243	101.3	100.8	101.9	364,187	370.2	368.9	371.4

CI, confidence interval (95%).

Table S4: Adjusted rate ratios of receiving a positive test for SARS-CoV-2 by sociodemographic characteristics and wave of the pandemic

		Wave two (1 September 2020 to 22 May 2021)			Wave three (23 May 2021 onwards)		
**Exposure**	Group	RR (Model 1)	RR (Model 2)	RR (Model 3)	RR (Model 1)	RR (Model 2)	RR (Model 3)
**Household tenure**	Owned	1 (ref)	1 (ref)	1 (ref)	1 (ref)	1 (ref)	1 (ref)
	Other	0.96 [0.95 - 0.97]	0.97 [0.96 - 0.98]	0.93 [0.92 - 0.94]	0.86 [0.85 - 0.86]	0.89 [0.88 - 0.89]	0.92 [0.91 - 0.93]
	Private rented	0.93 [0.93 - 0.93]	0.92 [0.92 - 0.92]	0.89 [0.89 - 0.90]	0.84 [0.84 - 0.84]	0.86 [0.86 - 0.86]	0.89 [0.89 - 0.90]
	Social rented	1.07 [1.07 - 1.07]	1.02 [1.02 - 1.02]	0.93 [0.93 - 0.93]	0.84 [0.84 - 0.84]	0.86 [0.86 - 0.86]	0.90 [0.90 - 0.90]
**Care home status**	No	1 (ref)	1 (ref)	1 (ref)	1 (ref)	1 (ref)	1 (ref)
	Yes	4.15 [4.11 - 4.20]	4.17 [4.13 - 4.22]	4.3 [4.25 - 4.35]	1.07 [1.04 - 1.10]	1.06 [1.03 - 1.09]	1.32 [1.28 - 1.36]
**National Statistics Socio-Economic Classification of the household reference person ***	1 Higher managerial, administrative and professional occupations	1 (ref)	1 (ref)	1 (ref)	1 (ref)	1 (ref)	1 (ref)
	2 Lower managerial, administrative and professional occupations	1.17 [1.16 - 1.18]	1.16 [1.15 - 1.16]	1.13 [1.12 - 1.13]	1.02 [1.02 - 1.02]	1.01 [1.01 - 1.01]	1.01 [1.01 - 1.01]
	3 Intermediate occupations	1.25 [1.24 - 1.26]	1.20 [1.20 - 1.21]	1.12 [1.11 - 1.13]	0.97 [0.97 - 0.98]	0.96 [0.95 - 0.96]	0.96 [0.96 - 0.97]
	4 Small employers and own account workers	1.29 [1.28 - 1.29]	1.29 [1.28 - 1.29]	1.15 [1.15 - 1.16]	0.92 [0.91 - 0.92]	0.91 [0.91 - 0.91]	0.95 [0.94 - 0.95]
	5 Lower supervisory and technical occupations	1.34 [1.33 - 1.35]	1.31 [1.30 - 1.32]	1.19 [1.18 - 1.19]	0.98 [0.98 - 0.98]	0.95 [0.94 - 0.95]	0.97 [0.97 - 0.97]
	6 Semi-routine occupations	1.39 [1.38 - 1.40]	1.33 [1.33 - 1.34]	1.18 [1.18 - 1.19]	0.92 [0.91 - 0.92]	0.89 [0.89 - 0.89]	0.93 [0.93 - 0.93]
	7 Routine occupations	1.38 [1.37 - 1.38]	1.32 [1.31 - 1.32]	1.16 [1.15 - 1.17]	0.92 [0.91 - 0.92]	0.88 [0.88 - 0.88]	0.93 [0.92 - 0.93]
	8 Never worked and long-term unemployed	1.27 [1.27 - 1.28]	1.15 [1.15 - 1.16]	0.97 [0.96 - 0.98]	0.74 [0.74 - 0.74]	0.74 [0.73 - 0.74]	0.81 [0.81 - 0.82]
**Country of birth**	UK	1 (ref)	1 (ref)	1 (ref)	1 (ref)	1 (ref)	1 (ref)
	Non-UK	1.21 [1.21 - 1.22]	1.14 [1.14 - 1.15]	1.05 [1.04 - 1.05]	0.70 [0.70 - 0.71]	0.77 [0.76 - 0.77]	0.96 [0.96 - 0.97]

		Wave two (1 September 2020 to 22 May 2021)			Wave three (23 May 2021 onwards)		
**English language proficiency**	Main language	1 (ref)	1 (ref)	1 (ref)			
	Well or very well	1.34 [1.34 - 1.35]	1.26 [1.25 - 1.26]	1.12 [1.11 - 1.12]	0.68 [0.67 - 0.68]	0.74 [0.73 - 0.74]	0.94 [0.93 - 0.94]
	Not well or not at all	1.48 [1.47 - 1.49]	1.35 [1.33 - 1.36]	1.10 [1.09 - 1.11]	0.58 [0.58 - 0.59]	0.62 [0.61 - 0.63]	0.83 [0.82 - 0.84]
**Rural-Urban Classification**	Villages, hamlets and isolated dwellings	1 (ref)	1 (ref)	1 (ref)	1 (ref)	1 (ref)	1 (ref)
	City and town	1.38 [1.37 - 1.38]	1.36 [1.35 - 1.37]	1.29 [1.28 - 1.29]	1.07 [1.06 - 1.07]	1.07 [1.07 - 1.07]	1.12 [1.11 - 1.12]
	Major or minor conurbation	1.80 [1.79 - 1.81]	1.60 [1.59 - 1.61]	1.46 [1.45 - 1.46]	0.95 [0.95 - 0.95]	1.02 [1.01 - 1.02]	1.11 [1.10 - 1.11]
	Town and fringe	1.18 [1.17 - 1.19]	1.15 [1.15 - 1.16]	1.15 [1.14 - 1.16]	1.09 [1.08 - 1.09]	1.08 [1.07 - 1.08]	1.08 [1.07 - 1.08]

RR, rate ratio; CI, confidence interval (95%).

Model 1, adjusted for age and sex only; Model 2, plus geography (region and Rural-Urban Classification); Model 3, fully-adjusted model. Note that for Religion the fully adjusted model (model 3) does not adjust for ethnicity.

* For the National Statistics Socio-Economic classification of the household reference person, model 3 does not adjust for household tenure, due to strong collinearity affecting convergence of the models.

Table S5: Adjusted rate ratios of receiving a positive test for SARS-CoV-2 by sociodemographic characteristics and broad age group during the second wave (1 September 2020 to 22 May 2021)

		Under 65			65+		
Exposure	Group	RR (Model 1)	RR (Model 2)	RR (Model 3)	RR (Model 1)	RR (Model 2)	RR (Model 3)
Disability status	Not Limited	1 (ref)	1 (ref)	1 (ref)	1 (ref)	1 (ref)	1 (ref)
	Limited a little	0.91 [0.9 - 0.91]	0.89 [0.89 - 0.9]	0.85 [0.85 - 0.86]	1.39 [1.38 - 1.4]	1.36 [1.34 - 1.37]	1.14 [1.13 - 1.15]
	Limited a lot	0.84 [0.83 - 0.84]	0.81 [0.8 - 0.82]	0.75 [0.74 - 0.76]	1.94 [1.93 - 1.96]	1.82 [1.81 - 1.84]	1.28 [1.27 - 1.3]
Ethnicity	White British	1 (ref)	1 (ref)	1 (ref)	1 (ref)	1 (ref)	1 (ref)
	Bangladeshi	1.97 [1.95 - 1.99]	1.79 [1.77 - 1.81]	1.73 [1.71 - 1.75]	3.45 [3.31 - 3.59]	2.89 [2.78 - 3.02]	2.51 [2.41 - 2.62]
	Black African	1.13 [1.12 - 1.14]	1.04 [1.03 - 1.05]	1.04 [1.03 - 1.05]	1.67 [1.6 - 1.75]	1.42 [1.36 - 1.48]	1.36 [1.3 - 1.42]
	Black Caribbean	1.07 [1.06 - 1.09]	0.98 [0.97 - 1]	0.96 [0.94 - 0.97]	1.38 [1.34 - 1.43]	1.18 [1.14 - 1.21]	1.09 [1.05 - 1.13]
	Chinese	0.53 [0.51 - 0.54]	0.5 [0.48 - 0.51]	0.54 [0.52 - 0.55]	0.7 [0.65 - 0.76]	0.62 [0.58 - 0.67]	0.69 [0.64 - 0.74]
	Indian	1.55 [1.54 - 1.56]	1.44 [1.43 - 1.45]	1.46 [1.45 - 1.47]	1.93 [1.89 - 1.97]	1.66 [1.63 - 1.7]	1.73 [1.69 - 1.76]
	Mixed	1.09 [1.08 - 1.1]	1.04 [1.03 - 1.05]	1.04 [1.04 - 1.05]	1.33 [1.27 - 1.39]	1.22 [1.16 - 1.28]	1.16 [1.1 - 1.21]
	Other	1.38 [1.37 - 1.39]	1.29 [1.28 - 1.3]	1.3 [1.29 - 1.31]	1.76 [1.71 - 1.8]	1.53 [1.49 - 1.57]	1.55 [1.51 - 1.59]
	Pakistani	1.94 [1.93 - 1.95]	1.71 [1.7 - 1.72]	1.65 [1.64 - 1.66]	3.4 [3.32 - 3.48]	2.84 [2.77 - 2.9]	2.6 [2.54 - 2.66]
	White Other	0.99 [0.98 - 0.99]	0.96 [0.95 - 0.97]	0.99 [0.99 - 1]	1.14 [1.12 - 1.16]	1.06 [1.04 - 1.08]	1.04 [1.02 - 1.06]
Education level	No qualification	1 (ref)	1 (ref)	1 (ref)	1 (ref)	1 (ref)	1 (ref)
	Apprenticeship	1 [0.99 - 1.01]	1.06 [1.05 - 1.07]	1.1 [1.08 - 1.11]	0.77 [0.76 - 0.78]	0.8 [0.79 - 0.81]	0.93 [0.91 - 0.94]
	Level 1	0.98 [0.98 - 0.99]	1.02 [1.02 - 1.03]	1.04 [1.04 - 1.05]	0.75 [0.74 - 0.76]	0.77 [0.76 - 0.79]	0.88 [0.87 - 0.89]
	Level 2	0.97 [0.97 - 0.98]	1.02 [1.01 - 1.03]	1.05 [1.04 - 1.06]	0.72 [0.71 - 0.73]	0.76 [0.75 - 0.77]	0.87 [0.86 - 0.89]
	Level 3	0.94 [0.94 - 0.95]	0.99 [0.98 - 0.99]	1.03 [1.02 - 1.03]	0.7 [0.69 - 0.71]	0.73 [0.72 - 0.75]	0.86 [0.85 - 0.87]
	Level 4	0.78 [0.78 - 0.79]	0.82 [0.81 - 0.82]	0.85 [0.84 - 0.85]	0.6 [0.59 - 0.6]	0.63 [0.63 - 0.64]	0.75 [0.74 - 0.76]
	Other	1.08 [1.08 - 1.09]	1.08 [1.07 - 1.09]	1.06 [1.05 - 1.06]	0.93 [0.92 - 0.94]	0.93 [0.92 - 0.94]	0.95 [0.93 - 0.96]

English Indices of Deprivation quintile group	1 (most deprived)	1.39 [1.38 - 1.39]	1.21 [1.21 - 1.22]	1.14 [1.14 - 1.15]	1.95 [1.93 - 1.97]	1.72 [1.71 - 1.74]	1.34 [1.32 - 1.35]
	2	1.25 [1.25 - 1.26]	1.17 [1.16 - 1.17]	1.12 [1.12 - 1.13]	1.5 [1.49 - 1.52]	1.44 [1.42 - 1.45]	1.22 [1.2 - 1.23]
	3	1.12 [1.12 - 1.13]	1.11 [1.11 - 1.12]	1.09 [1.08 - 1.09]	1.23 [1.21 - 1.24]	1.26 [1.25 - 1.28]	1.14 [1.13 - 1.15]
	4	1.08 [1.07 - 1.08]	1.08 [1.07 - 1.08]	1.06 [1.06 - 1.07]	1.11 [1.1 - 1.12]	1.13 [1.12 - 1.15]	1.08 [1.06 - 1.09]
	5 (least deprived)	1 (ref)	1 (ref)	1 (ref)	1 (ref)	1 (ref)	1 (ref)
Religion	Christian	1 (ref)	1 (ref)	1 (ref)	1 (ref)	1 (ref)	1 (ref)
	Buddhist	0.8 [0.78 - 0.82]	0.8 [0.78 - 0.82]	0.84 [0.82 - 0.86]	0.81 [0.76 - 0.87]	0.77 [0.72 - 0.83]	0.83 [0.77 - 0.89]
	Hindu	1.24 [1.23 - 1.25]	1.18 [1.17 - 1.2]	1.21 [1.2 - 1.23]	1.59 [1.55 - 1.63]	1.36 [1.33 - 1.4]	1.45 [1.41 - 1.49]
	Jewish	1.07 [1.04 - 1.09]	1 [0.98 - 1.02]	1.03 [1.01 - 1.06]	1.1 [1.06 - 1.15]	0.95 [0.91 - 0.99]	1.06 [1.02 - 1.11]
	Muslim	1.66 [1.65 - 1.67]	1.52 [1.51 - 1.53]	1.48 [1.47 - 1.49]	2.82 [2.78 - 2.87]	2.38 [2.34 - 2.42]	2.19 [2.15 - 2.23]
	Sikh	1.71 [1.69 - 1.73]	1.61 [1.59 - 1.63]	1.59 [1.58 - 1.61]	2.3 [2.23 - 2.37]	1.99 [1.93 - 2.06]	2.04 [1.98 - 2.1]
	No religion	0.85 [0.85 - 0.85]	0.87 [0.87 - 0.87]	0.88 [0.88 - 0.88]	0.79 [0.78 - 0.8]	0.81 [0.8 - 0.82]	0.85 [0.84 - 0.86]
	Other religion	0.77 [0.75 - 0.78]	0.78 [0.77 - 0.8]	0.8 [0.78 - 0.82]	0.92 [0.87 - 0.98]	0.92 [0.87 - 0.98]	0.95 [0.9 - 1.01]
	Not stated	0.86 [0.86 - 0.87]	0.87 [0.87 - 0.88]	0.88 [0.87 - 0.88]	0.94 [0.93 - 0.95]	0.95 [0.94 - 0.96]	0.94 [0.93 - 0.96]
Household tenure	Owned	1 (ref)	1 (ref)	1 (ref)	1 (ref)	1 (ref)	1 (ref)
	Other	0.91 [0.9 - 0.92]	0.93 [0.92 - 0.94]	0.9 [0.89 - 0.91]	1.28 [1.25 - 1.32]	1.28 [1.25 - 1.31]	1.09 [1.07 - 1.12]
	Private rented	0.9 [0.9 - 0.91]	0.9 [0.89 - 0.9]	0.88 [0.88 - 0.89]	1.25 [1.23 - 1.27]	1.26 [1.24 - 1.28]	1.09 [1.07 - 1.1]
	Social rented	1.01 [1.01 - 1.02]	0.97 [0.96 - 0.97]	0.91 [0.91 - 0.91]	1.53 [1.52 - 1.54]	1.41 [1.4 - 1.43]	1.11 [1.1 - 1.12]
Care home status	No	1 (ref)	1 (ref)	1 (ref)	1 (ref)	1 (ref)	1 (ref)
	Yes	2.43 [2.36 - 2.49]	2.51 [2.44 - 2.57]	3 [2.92 - 3.08]	5.12 [5.05 - 5.18]	5.09 [5.02 - 5.15]	4.42 [4.36 - 4.48]
National Statistics Socio-Economic Classification of the household	1 Higher managerial, administrative and professional occupations	1 (ref)	1 (ref)	1 (ref)	1 (ref)	1 (ref)	1 (ref)
	2 Lower managerial, administrative and professional occupations	1.17 [1.17 - 1.18]	1.16 [1.15 - 1.17]	1.13 [1.13 - 1.14]	1.19 [1.17 - 1.2]	1.17 [1.15 - 1.18]	1.1 [1.09 - 1.12]

**reference person***	3 Intermediate occupations	1.25 [1.24 - 1.26]	1.21 [1.2 - 1.21]	1.14 [1.13 - 1.14]	1.32 [1.3 - 1.34]	1.26 [1.24 - 1.28]	1.1 [1.08 - 1.12]
	4 Small employers and own account workers	1.28 [1.27 - 1.29]	1.28 [1.27 - 1.28]	1.16 [1.15 - 1.16]	1.36 [1.34 - 1.38]	1.38 [1.36 - 1.4]	1.16 [1.15 - 1.18]
	5 Lower supervisory and technical occupations	1.32 [1.31 - 1.33]	1.3 [1.29 - 1.3]	1.19 [1.18 - 1.2]	1.49 [1.47 - 1.52]	1.42 [1.4 - 1.44]	1.17 [1.15 - 1.19]
	6 Semi-routine occupations	1.37 [1.36 - 1.37]	1.31 [1.31 - 1.32]	1.19 [1.18 - 1.2]	1.6 [1.57 - 1.62]	1.51 [1.49 - 1.53]	1.19 [1.17 - 1.2]
	7 Routine occupations	1.33 [1.32 - 1.33]	1.27 [1.27 - 1.28]	1.15 [1.15 - 1.16]	1.71 [1.69 - 1.73]	1.59 [1.57 - 1.62]	1.2 [1.19 - 1.22]
	8 Never worked and long-term unemployed	1.21 [1.2 - 1.22]	1.1 [1.09 - 1.11]	0.97 [0.96 - 0.97]	2 [1.96 - 2.04]	1.8 [1.76 - 1.84]	1.17 [1.14 - 1.19]
**Country of birth**	UK	1 (ref)	1 (ref)	1 (ref)	1 (ref)	1 (ref)	1 (ref)
	Non-UK	1.17 [1.17 - 1.18]	1.11 [1.11 - 1.12]	1.02 [1.01 - 1.02]	1.51 [1.49 - 1.52]	1.36 [1.35 - 1.37]	1 [0.98 - 1.02]
**English language proficiency**	Main language	1 (ref)	1 (ref)	1 (ref)	1 (ref)	1 (ref)	1 (ref)
	Well or very well	1.31 [1.3 - 1.31]	1.23 [1.22 - 1.24]	1.11 [1.1 - 1.11]	1.86 [1.83 - 1.89]	1.63 [1.6 - 1.65]	1.16 [1.13 - 1.18]
	Not well or not at all	1.35 [1.34 - 1.37]	1.24 [1.23 - 1.26]	1.05 [1.04 - 1.06]	2.29 [2.25 - 2.34]	1.94 [1.9 - 1.98]	1.12 [1.09 - 1.15]
**Rural-Urban Classification**	Villages, hamlets and isolated dwellings	1 (ref)	1 (ref)	1 (ref)	1 (ref)	1 (ref)	1 (ref)
	City and town	1.35 [1.34 - 1.36]	1.33 [1.33 - 1.34]	1.28 [1.27 - 1.28]	1.5 [1.48 - 1.52]	1.47 [1.45 - 1.49]	1.32 [1.3 - 1.34]
	Major or minor conurbation	1.75 [1.74 - 1.76]	1.57 [1.56 - 1.58]	1.44 [1.43 - 1.45]	2.06 [2.03 - 2.09]	1.77 [1.74 - 1.79]	1.49 [1.47 - 1.51]
	Town and fringe	1.18 [1.17 - 1.19]	1.15 [1.14 - 1.16]	1.15 [1.14 - 1.16]	1.19 [1.17 - 1.21]	1.16 [1.14 - 1.18]	1.14 [1.12 - 1.16]

RR, rate ratio; CI, confidence interval (95%).
Model 1, adjusted for age and sex only; Model 2, plus geography (region and Rural-Urban Classification); Model 3, fully-adjusted model. Note that for Religion the fully adjusted model (model 3) does not adjust for ethnicity.

* For the National Statistics Socio-Economic classification of the household reference person, model 3 does not adjust for household tenure, due to strong collinearity affecting convergence of the models.

Table S6: Adjusted rate ratios of receiving a positive test for SARS-CoV-2 by sociodemographic characteristics and broad age group during the third wave (23 May 2021 to 10 December 2021)

		Under 65			65+		
**Exposure**	Group	RR (Model 1)	RR (Model 2)	RR (Model 3)	RR (Model 1)	RR (Model 2)	RR (Model 3)
**Disability status**	Not Limited	1 (ref)	1 (ref)	1 (ref)	1 (ref)	1 (ref)	1 (ref)
	Limited a little	0.81 [0.80 - 0.81]	0.80 [0.80 - 0.81]	0.83 [0.83 - 0.84]	1.06 [1.05 - 1.07]	1.03 [1.02 - 1.04]	0.97 [0.96 - 0.98]
	Limited a lot	0.62 [0.62 - 0.63]	0.62 [0.61 - 0.62]	0.68 [0.67 - 0.68]	1.18 [1.16 - 1.19]	1.11 [1.10 - 1.13]	1.00 [0.99 - 1.01]
**Ethnicity**	White British	1 (ref)	1 (ref)	1 (ref)	1 (ref)	1 (ref)	1 (ref)
	Bangladeshi	0.58 [0.57 - 0.58]	0.63 [0.63 - 0.64]	0.66 [0.66 - 0.67]	1.48 [1.39 - 1.59]	1.63 [1.52 - 1.74]	1.61 [1.50 - 1.72]
	Black African	0.54 [0.54 - 0.55]	0.61 [0.60 - 0.61]	0.64 [0.63 - 0.65]	0.69 [0.65 - 0.74]	0.81 [0.75 - 0.86]	0.81 [0.75 - 0.87]
	Black Caribbean	0.77 [0.76 - 0.77]	0.86 [0.85 - 0.87]	0.90 [0.88 - 0.91]	0.98 [0.94 - 1.02]	1.08 [1.04 - 1.13]	1.07 [1.02 - 1.12]
	Chinese	0.45 [0.44 - 0.46]	0.48 [0.47 - 0.49]	0.49 [0.48 - 0.50]	0.41 [0.37 - 0.46]	0.44 [0.40 - 0.49]	0.48 [0.43 - 0.53]
	Indian	0.72 [0.72 - 0.73]	0.77 [0.77 - 0.78]	0.76 [0.76 - 0.77]	1.20 [1.17 - 1.23]	1.29 [1.26 - 1.33]	1.30 [1.26 - 1.33]
	Mixed	0.85 [0.84 - 0.86]	0.90 [0.89 - 0.90]	0.92 [0.92 - 0.93]	0.92 [0.86 - 0.98]	0.98 [0.92 - 1.04]	0.98 [0.92 - 1.04]
	Other	0.61 [0.61 - 0.62]	0.68 [0.67 - 0.68]	0.71 [0.70 - 0.71]	0.95 [0.92 - 0.98]	1.07 [1.03 - 1.11]	1.09 [1.05 - 1.13]
	Pakistani	0.59 [0.58 - 0.59]	0.59 [0.59 - 0.60]	0.61 [0.60 - 0.61]	1.44 [1.38 - 1.49]	1.38 [1.33 - 1.43]	1.32 [1.27 - 1.37]
	White Other	0.72 [0.72 - 0.73]	0.78 [0.78 - 0.79]	0.82 [0.82 - 0.83]	0.89 [0.87 - 0.91]	0.97 [0.95 - 0.99]	0.97 [0.95 – 1.00]
**Education level**	No qualification	1 (ref)	1 (ref)	1 (ref)	1 (ref)	1 (ref)	1 (ref)
	Apprenticeship	1.37 [1.35 - 1.38]	1.34 [1.32 - 1.35]	1.21 [1.20 - 1.22]	1.02 [1 - 1.04]	1.02 [1.01 - 1.04]	1.05 [1.03 - 1.07]
	Level 1	1.23 [1.22 - 1.24]	1.22 [1.22 - 1.23]	1.15 [1.14 - 1.15]	0.96 [0.94 - 0.97]	0.99 [0.98 - 1.01]	1.02 [1.01 - 1.04]
	Level 2	1.27 [1.27 - 1.28]	1.27 [1.26 - 1.27]	1.16 [1.16 - 1.17]	0.94 [0.93 - 0.95]	0.97 [0.96 - 0.98]	1.01 [0.99 - 1.02]
	Level 3	1.30 [1.29 - 1.31]	1.29 [1.29 - 1.30]	1.19 [1.18 - 1.19]	0.95 [0.94 - 0.97]	0.99 [0.97 – 1.00]	1.02 [1.01 - 1.04]
	Level 4	1.26 [1.25 - 1.27]	1.30 [1.29 - 1.31]	1.20 [1.20 - 1.21]	0.88 [0.87 - 0.89]	0.93 [0.92 - 0.94]	0.98 [0.97 - 0.99]

	Other	0.94 [0.93 - 0.95]	1.00 [0.99 - 1.01]	1.08 [1.07 - 1.09]	0.99 [0.97 – 1.00]	1.02 [1 - 1.04]	1.03 [1.01 - 1.04]
**English Indices of Deprivation quintile group**	1 (most deprived)	0.85 [0.85 - 0.86]	0.82 [0.82 - 0.82]	0.92 [0.92 - 0.92]	1.29 [1.27 - 1.3]	1.12 [1.11 - 1.14]	1.09 [1.08 - 1.11]
	2	0.88 [0.88 - 0.88]	0.89 [0.89 - 0.90]	0.96 [0.95 - 0.96]	1.15 [1.14 - 1.17]	1.11 [1.09 - 1.12]	1.08 [1.07 - 1.10]
	3	0.92 [0.92 - 0.92]	0.94 [0.93 - 0.94]	0.97 [0.97 - 0.97]	1.05 [1.03 - 1.06]	1.05 [1.04 - 1.07]	1.04 [1.02 - 1.05]
	4	0.97 [0.96 - 0.97]	0.97 [0.96 - 0.97]	0.98 [0.98 - 0.99]	1.05 [1.03 - 1.06]	1.04 [1.03 - 1.06]	1.03 [1.02 - 1.05]
	5 (least deprived)	1 (ref)	1 (ref)	1 (ref)	1 (ref)	1 (ref)	1 (ref)
**Religion**	Christian	1 (ref)	1 (ref)	1 (ref)	1 (ref)	1 (ref)	1 (ref)
	Buddhist	0.64 [0.62 - 0.65]	0.68 [0.66 - 0.69]	0.71 [0.69 - 0.72]	0.59 [0.54 - 0.65]	0.65 [0.59 - 0.70]	0.68 [0.62 - 0.74]
	Hindu	0.74 [0.73 - 0.75]	0.83 [0.82 - 0.84]	0.82 [0.82 - 0.83]	1.13 [1.10 - 1.17]	1.27 [1.22 - 1.31]	1.28 [1.24 - 1.33]
	Jewish	0.82 [0.80 - 0.83]	0.94 [0.92 - 0.96]	0.92 [0.9 - 0.94]	1.06 [1.01 - 1.12]	1.19 [1.14 - 1.26]	1.22 [1.16 - 1.28]
	Muslim	0.59 [0.59 - 0.60]	0.63 [0.63 - 0.64]	0.66 [0.66 - 0.66]	1.24 [1.21 - 1.27]	1.28 [1.25 - 1.31]	1.25 [1.22 - 1.29]
	Sikh	0.79 [0.78 - 0.80]	0.83 [0.82 - 0.85]	0.82 [0.81 - 0.83]	1.32 [1.27 - 1.38]	1.39 [1.33 - 1.45]	1.38 [1.32 - 1.44]
	No religion	0.96 [0.96 - 0.96]	0.96 [0.96 - 0.96]	0.98 [0.97 - 0.98]	0.83 [0.82 - 0.84]	0.85 [0.84 - 0.86]	0.87 [0.86 - 0.88]
	Other religion	0.76 [0.75 - 0.78]	0.78 [0.77 - 0.80]	0.79 [0.78 - 0.81]	0.91 [0.86 - 0.97]	0.97 [0.91 - 1.03]	0.97 [0.92 - 1.03]
	Not stated	0.87 [0.87 - 0.88]	0.89 [0.88 - 0.89]	0.90 [0.89 - 0.90]	0.84 [0.82 - 0.85]	0.86 [0.84 - 0.87]	0.86 [0.85 - 0.88]
**Household tenure**	Owned	1 (ref)	1 (ref)	1 (ref)	1 (ref)	1 (ref)	1 (ref)
	Other	0.85 [0.84 - 0.85]	0.88 [0.87 - 0.89]	0.92 [0.91 - 0.92]	0.98 [0.94 - 1.01]	0.99 [0.96 - 1.02]	0.95 [0.92 - 0.99]
	Private rented	0.84 [0.84 - 0.84]	0.86 [0.86 - 0.86]	0.90 [0.89 - 0.90]	0.93 [0.91 - 0.95]	0.95 [0.93 - 0.96]	0.91 [0.89 - 0.92]
	Social rented	0.83 [0.83 - 0.83]	0.85 [0.85 - 0.86]	0.90 [0.90 - 0.91]	1.01 [1.00 - 1.02]	0.98 [0.97 - 0.99]	0.91 [0.90 - 0.92]
**Care home status**	No	1 (ref)	1 (ref)	1 (ref)	1 (ref)	1 (ref)	1 (ref)
	Yes	0.58 [0.55 - 0.61]	0.57 [0.54 - 0.60]	0.84 [0.80 - 0.88]	1.72 [1.67 - 1.78]	1.69 [1.64 - 1.75]	1.65 [1.59 - 1.70]
**National Statistics Socio-Economic**	1 Higher managerial, administrative and professional occupations	1 (ref)	1 (ref)	1 (ref)	1 (ref)	1 (ref)	1 (ref)

**Classification of the household reference person***	2 Lower managerial, administrative and professional occupations	1.02 [1.01 - 1.02]	1.01 [1.00 - 1.01]	1.01 [1.01 - 1.02]	1.05 [1.04 - 1.07]	1.03 [1.02 - 1.05]	1.02 [1.00 - 1.03]
	3 Intermediate occupations	0.97 [0.97 - 0.98]	0.96 [0.95 - 0.96]	0.98 [0.98 - 0.99]	1.05 [1.03 - 1.06]	1.01 [0.99 - 1.03]	0.97 [0.96 - 0.99]
	4 Small employers and own account workers	0.91 [0.9 - 0.91]	0.9 [0.9 - 0.9]	0.95 [0.95 - 0.95]	1.06 [1.05 - 1.08]	1.06 [1.04 - 1.08]	1.01 [1 - 1.03]
	5 Lower supervisory and technical occupations	0.97 [0.97 - 0.97]	0.94 [0.93 - 0.94]	0.98 [0.97 - 0.98]	1.13 [1.11 - 1.15]	1.06 [1.04 - 1.08]	1 [0.98 - 1.02]
	6 Semi-routine occupations	0.91 [0.9 - 0.91]	0.88 [0.87 - 0.88]	0.95 [0.95 - 0.96]	1.11 [1.1 - 1.13]	1.05 [1.03 - 1.06]	0.98 [0.97 - 1]
	7 Routine occupations	0.9 [0.89 - 0.9]	0.87 [0.86 - 0.87]	0.94 [0.94 - 0.95]	1.15 [1.14 - 1.17]	1.06 [1.05 - 1.08]	0.99 [0.97 - 1]
	8 Never worked and long-term unemployed	0.73 [0.72 - 0.73]	0.73 [0.73 - 0.73]	0.86 [0.85 - 0.86]	1.01 [0.99 - 1.04]	0.96 [0.93 - 0.99]	0.86 [0.84 - 0.89]
**Country of birth**	UK	1 (ref)	1 (ref)	1 (ref)	1 (ref)	1 (ref)	1 (ref)
	Non-UK	0.68 [0.68 - 0.68]	0.74 [0.74 - 0.75]	0.94 [0.93 - 0.94]	0.97 [0.96 - 0.98]	1.06 [1.04 - 1.07]	0.95 [0.92 - 0.97]
**English language proficiency**	Main language	1 (ref)	1 (ref)	1 (ref)	1 (ref)	1 (ref)	1 (ref)
	Well or very well	0.66 [0.66 - 0.67]	0.72 [0.72 - 0.73]	0.92 [0.92 - 0.93]	1.05 [1.02 - 1.07]	1.13 [1.11 - 1.16]	1.02 [0.99 - 1.06]
	Not well or not at all	0.54 [0.54 - 0.55]	0.58 [0.58 - 0.59]	0.79 [0.78 - 0.8]	1.17 [1.13 - 1.2]	1.2 [1.17 - 1.24]	1.05 [1.01 - 1.09]
**Rural-Urban Classification**	Villages, hamlets and isolated dwellings	1 (ref)	1 (ref)	1 (ref)	1 (ref)	1 (ref)	1 (ref)
	City and town	1.05 [1.04 - 1.05]	1.05 [1.05 - 1.05]	1.1 [1.1 - 1.11]	1.24 [1.22 - 1.26]	1.24 [1.23 - 1.26]	1.21 [1.2 - 1.23]
	Major or minor conurbation	0.92 [0.91 - 0.92]	0.99 [0.99 - 1]	1.09 [1.08 - 1.09]	1.35 [1.34 - 1.37]	1.34 [1.32 - 1.36]	1.29 [1.27 - 1.31]
	Town and fringe	1.08 [1.07 - 1.08]	1.07 [1.06 - 1.07]	1.07 [1.06 - 1.08]	1.16 [1.14 - 1.18]	1.14 [1.12 - 1.16]	1.13 [1.11 - 1.15]

RR, rate ratio; CI, confidence interval (95%).
Model 1, adjusted for age and sex only; Model 2, plus geography (region and Rural-Urban Classification); Model 3, fully-adjusted model. Note that for Religion the fully adjusted model (model 3) does not adjust for ethnicity.

* For the National Statistics Socio-Economic classification of the household reference person, model 3 does not adjust for household tenure, due to strong collinearity affecting convergence of the models.

Plot S1: Rate ratios by wave of the pandemic – Geographical variables

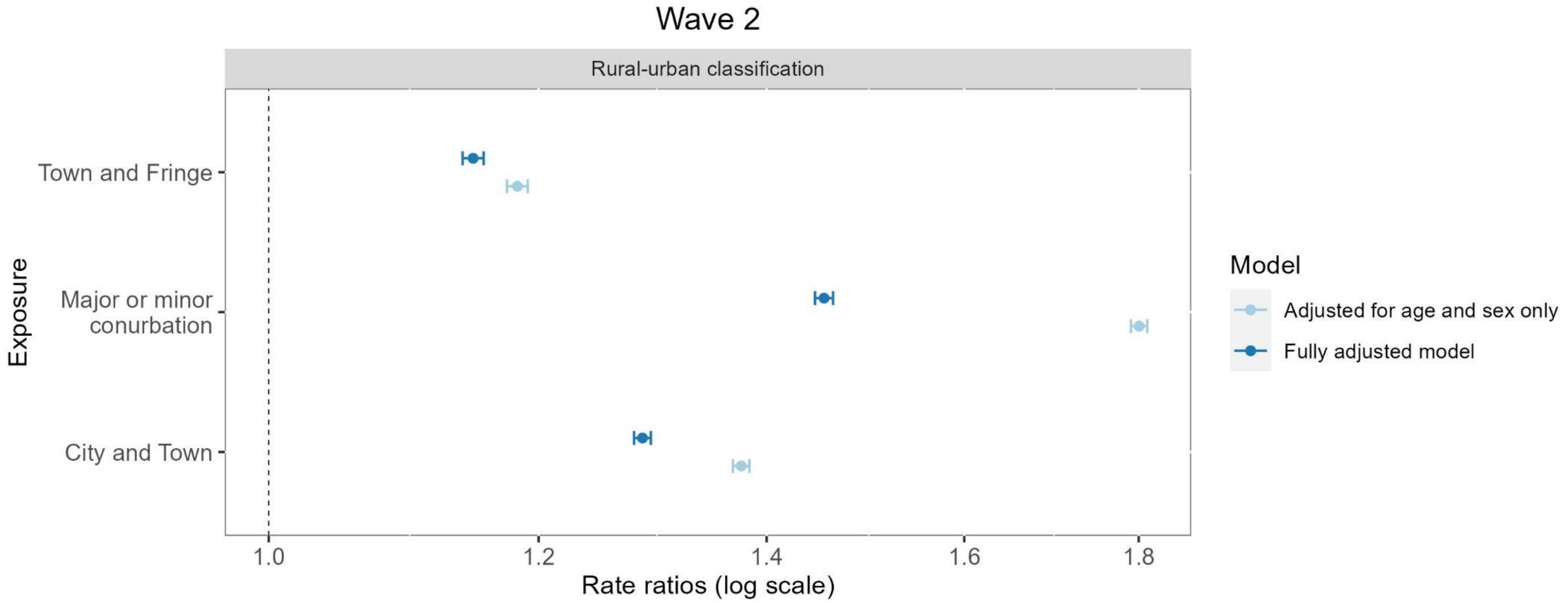

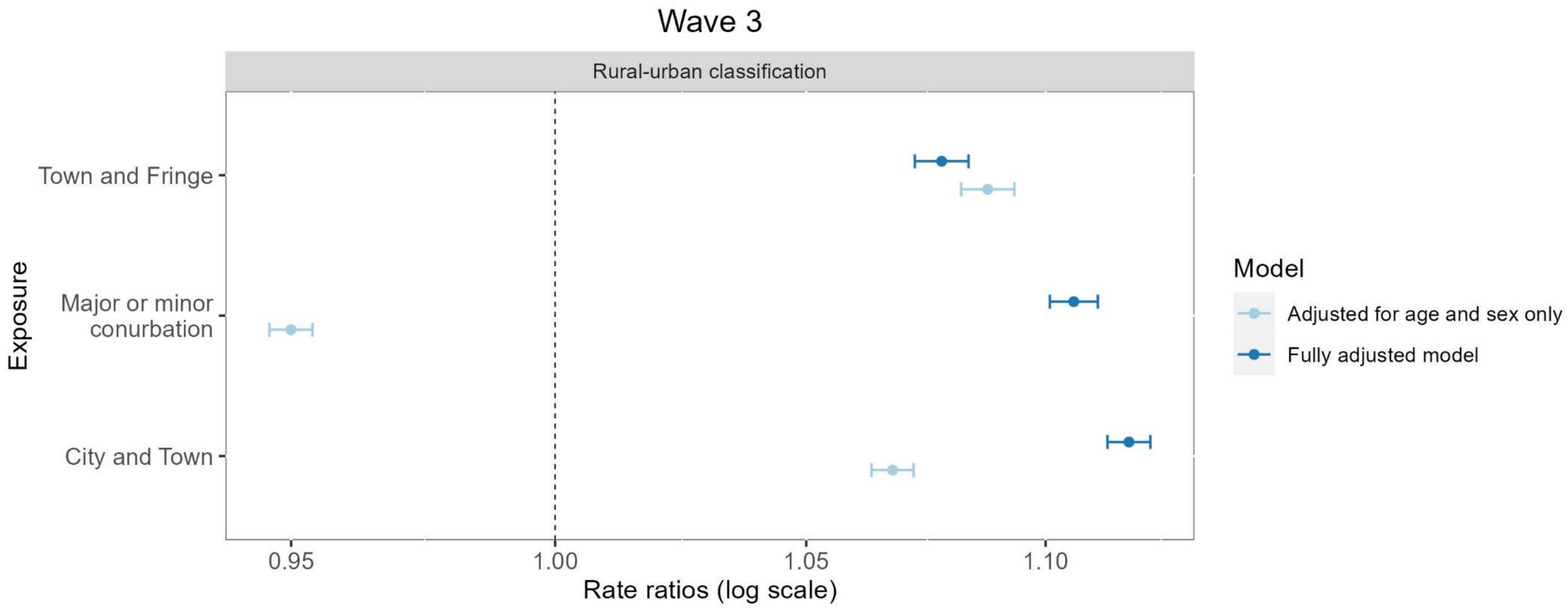
Wave 3
Rural-urban classification
Exposure
Town and Fringe
Major or minor conurbation
City and Town
0.95
1.00
1.05
1.10
Rate ratios (log scale)
Model
Adjusted for age and sex only
Fully adjusted model

Plot S2: Rate ratios by wave of the pandemic – sociodemographic variables

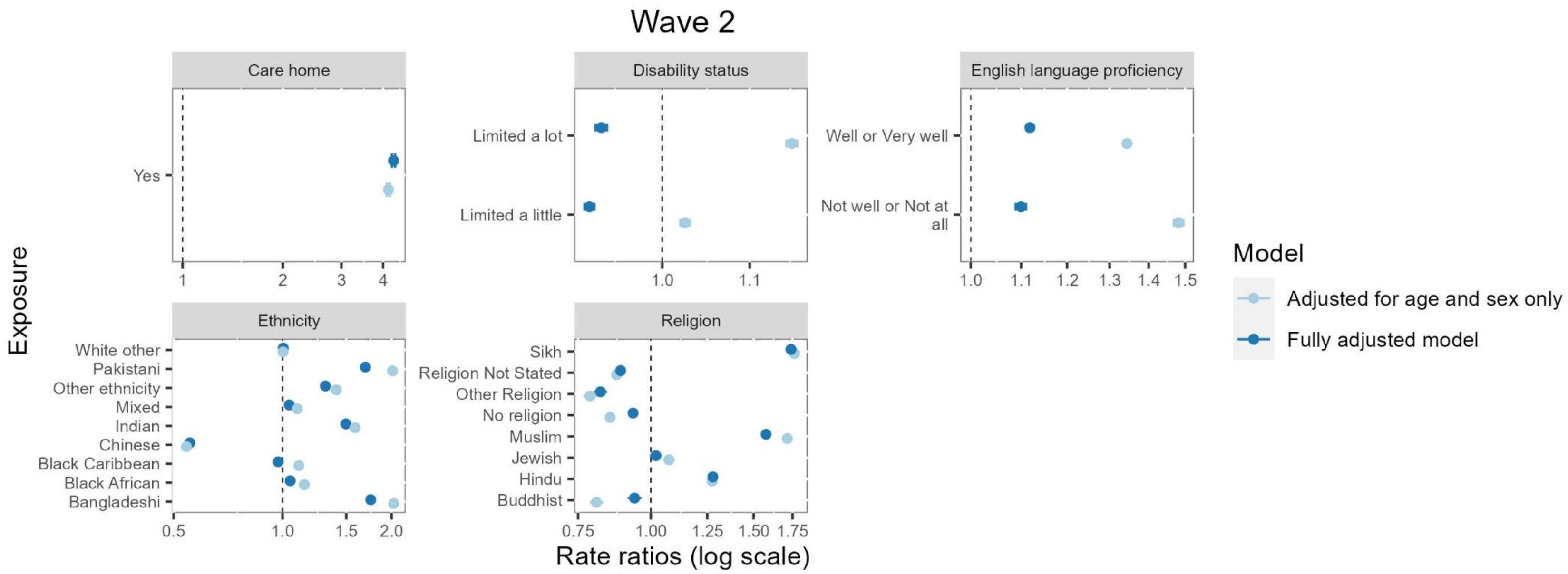

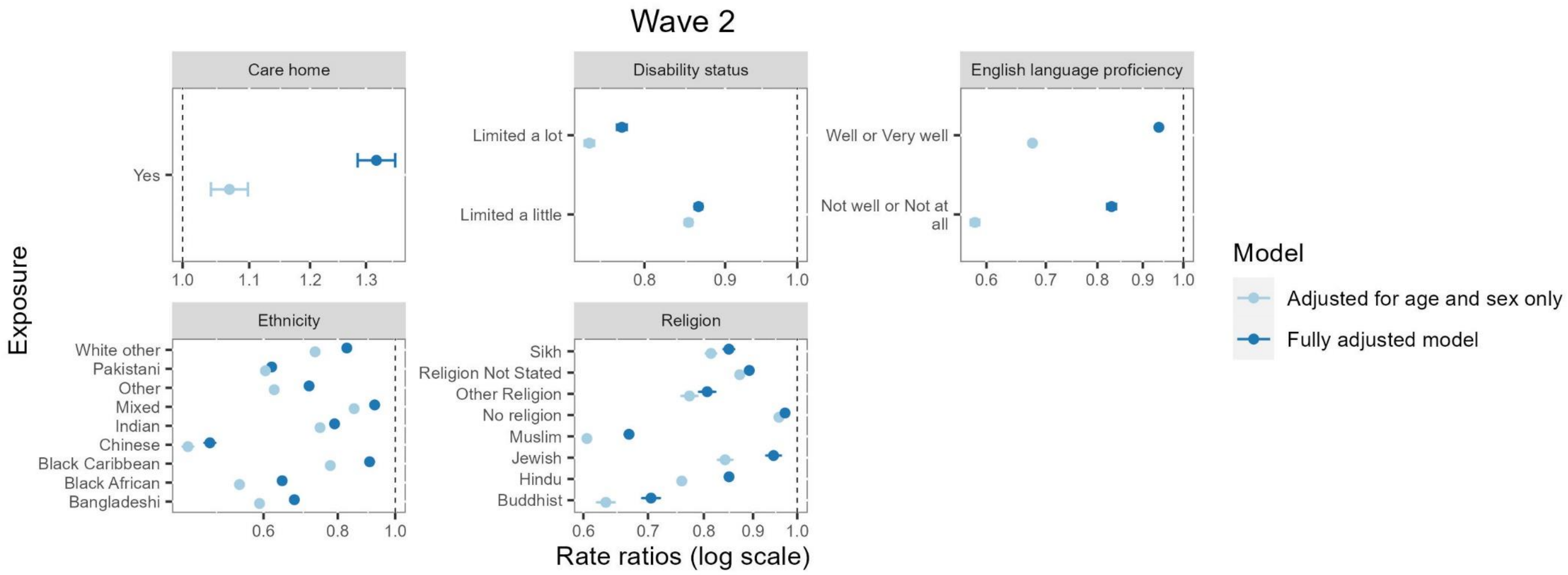

For Religion, the fully adjusted model does not adjust for ethnicity.

Plot S3: Rate ratios by wave of the pandemic – socioeconomic variables

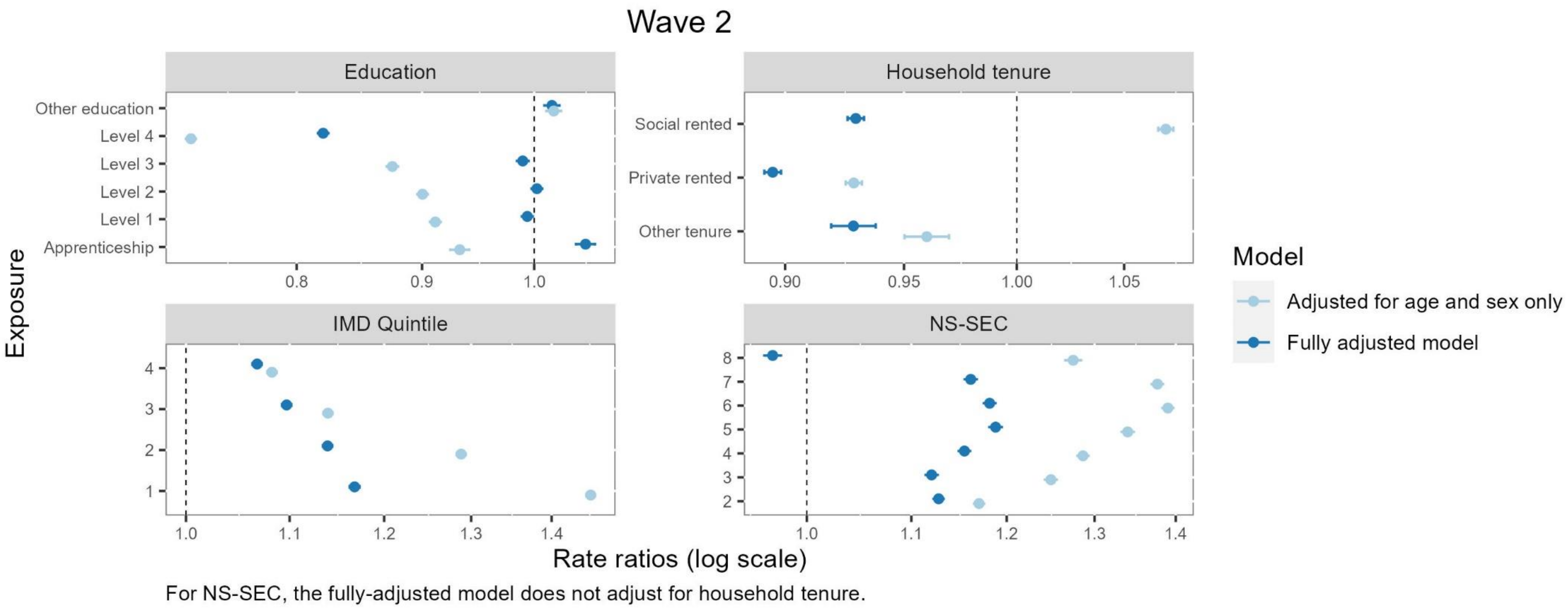

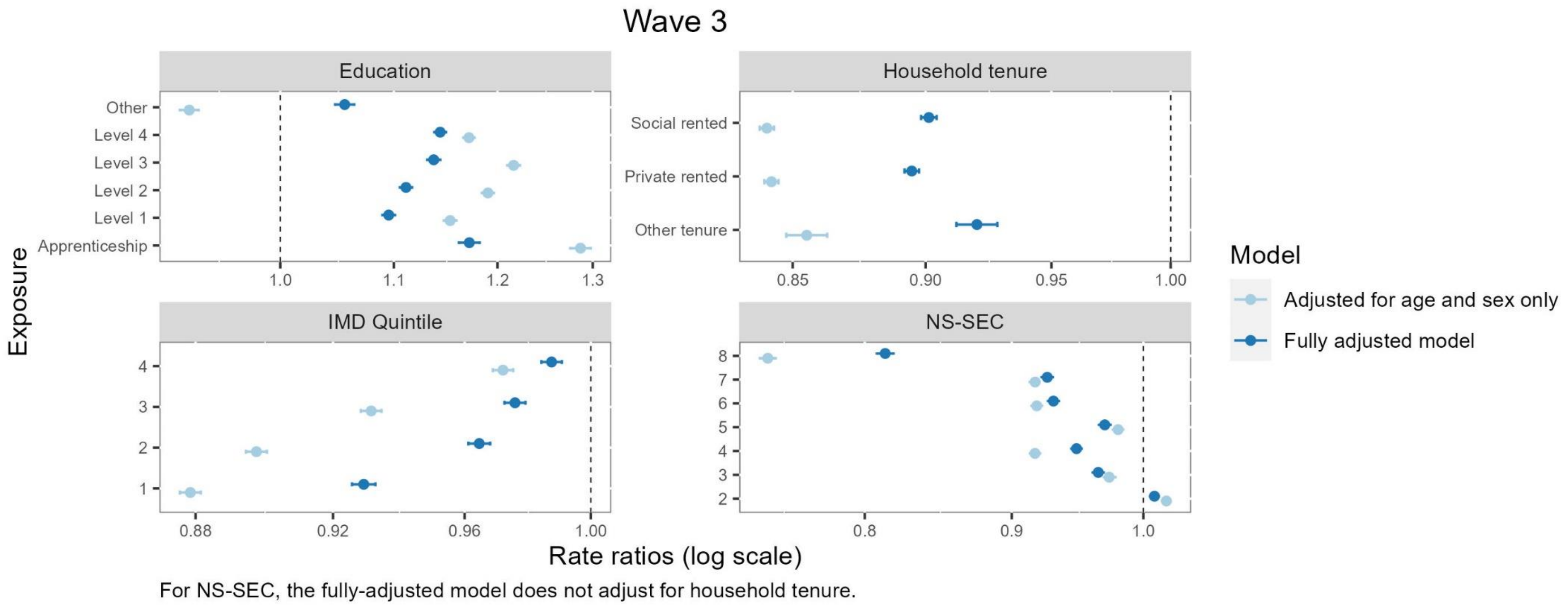

* For the National Statistics Socio-Economic classification (NS-SEC) of the household reference person, the fully-adjusted model does not adjust for household tenure, due to strong collinearity affecting convergence of the models. Please see Table S2 for a look-up of the NS-SEC group which corresponds to the given number (omitted within the plot due to text length).
