# Supplementary material for: Inequalities in SARS-CoV-2 case rates by ethnicity, religion, measures of socioeconomic position, English proficiency, and self-reported disability: cohort study of 39 million people in England during the alpha and delta waves
Source: BMJ Med. 2023 Apr 3;2(1):e000187. doi: 10.1136/bmjmed-2022-000187 (PMC10568121; doi:10.1136/bmjmed-2022-000187)
Supplement: Supplementary data [file bmjmed-2022-000187supp002.pdf]

term	p.value	rr	lower_ci	upper_ci
(Intercept)	0.981382	0.953584	0.017607	51.6443
age_1_1	2.67E-07	0.135376	0.063203	0.289964
age_1_2	0.00029	0.406031	0.249381	0.661081
age_1_3	2.52E-05	0.476776	0.337794	0.672939
age_1_4	2.40E-05	0.56123	0.42927	0.733756
age_1_5	1.17E-06	0.578988	0.464471	0.721739
age_1_6	8.33E-05	0.68196	0.563573	0.825215
age_1_7	0.781074	1.025131	0.860517	1.221234
age_1_8	1.13E-08	0.611793	0.516839	0.724191
age_1_9	2.62E-14	0.468773	0.385733	0.569689
age_2_1	1.23E-08	1.149051	1.095407	1.205322
age_2_2	0.001656	1.03195	1.011926	1.052371
age_2_3	3.83E-05	1.021041	1.010971	1.031211
age_2_4	4.15E-05	1.012697	1.006605	1.018825
age_2_5	6.67E-08	1.011406	1.007251	1.015578
age_2_6	0.000147	1.006192	1.00299	1.009404
age_2_7	3.05E-05	0.993947	0.991115	0.996788
age_2_8	7.99E-07	1.006917	1.004164	1.009677
age_2_9	3.68E-13	1.012726	1.009278	1.016186
age_3_1	3.53E-09	0.997014	0.996025	0.998004
age_3_2	0.00499	0.999628	0.999368	0.999888
age_3_3	5.39E-05	0.999804	0.999709	0.999899
age_3_4	6.96E-05	0.999908	0.999862	0.999953
age_3_5	2.06E-09	0.999921	0.999895	0.999947
age_3_6	3.46E-05	0.999961	0.999943	0.99998
age_3_7	3.93E-11	1.000053	1.000037	1.000069
age_3_8	0.000108	0.999972	0.999957	0.999986
age_3_9	1.36E-11	0.999941	0.999923	0.999958
sex_1	0	0.911145	0.907709	0.914594
region_1	0	1.077653	1.067799	1.087598
region_2	0	1.057706	1.05053	1.064931
region_3	0	0.70705	0.700732	0.713425
region_4	0	1.19457	1.186926	1.202262
region_5	0	1.065165	1.054419	1.076019
region_6	0	1.065358	1.057336	1.07344
region_7	4.60E-13	1.033236	1.024127	1.042427
region_8	0	1.098494	1.087718	1.109377
ruralurban	0	1.168276	1.155794	1.180893
ruralurban	0	1.501075	1.487736	1.514534
ruralurban	0	1.308319	1.297323	1.319407
bmi_categ	0	1.29785	1.280619	1.315314
bmi_categ	0	1.129059	1.11414	1.144177
bmi_categ	0	1.453731	1.434403	1.473319
health_cor	0	1.065634	1.063442	1.067832
learning_c	0	1.121053	1.102461	1.139959
ethnicity_	1.83E-05	0.971029	0.958058	0.984175
ethnicity_	0	1.71739	1.566244	1.883121
ethnicity_	0	1.154881	1.131914	1.178314
ethnicity_	0.618673	1.004883	0.985793	1.024342

ethnicity_5	0	1.438907	1.395687	1.483465
ethnicity_6	0	0.656147	0.610354	0.705377
ethnicity_7	0.010294	0.977574	0.960781	0.994659
ethnicity_8	2.69E-06	1.040162	1.023196	1.057409
ethnicity_9	0.004334	1.450309	1.123386	1.872372
imd_quint	0	1.084393	1.077715	1.091112
imd_quint	0	1.128085	1.121138	1.135074
imd_quint	0	1.172709	1.165489	1.179974
imd_quint	0	1.211917	1.204048	1.219838
religion_1	0.047245	1.20049	1.002222	1.437982
religion_2	0	0.869482	0.861473	0.877565
religion_3	0	0.697809	0.671885	0.724733
religion_4	0	0.883163	0.878747	0.887601
religion_5	0	1.331518	1.275015	1.390526
religion_6	0.516366	1.009294	0.981484	1.037893
religion_7	0.397491	0.91806	0.753116	1.119128
religion_8	0	0.700791	0.659415	0.744762
education_	0.691375	0.998137	0.988992	1.007368
education_	0.947247	0.999233	0.976786	1.022197
education_	0	0.847267	0.841813	0.852755
education_	0.102068	1.00611	0.99879	1.013484
education_	0.000725	1.011677	1.004889	1.018511
education_	0.473848	0.997503	0.990702	1.004351
education_	1.96E-11	1.042184	1.02968	1.05484
tenure_1	0	0.951765	0.946634	0.956924
tenure_2	0	0.914052	0.908953	0.919179
tenure_3	4.44E-16	0.94188	0.92845	0.955505
tenure_4	0	0.850101	0.834558	0.865934
care_home	0	4.19185	4.128999	4.255658
english_lar	0.001063	1.079401	1.031131	1.129931
english_lar	0.046677	0.919262	0.846089	0.998764
ethnicity_r	0.281484	1.196459	0.863243	1.6583
ethnicity_r	0.001413	0.942622	0.909034	0.977452
ethnicity_r	0.428755	1.056739	0.921728	1.211527
ethnicity_r	0	0.838152	0.817661	0.859156
ethnicity_r	0.329129	0.974582	0.925465	1.026305
ethnicity_r	0.190682	0.952216	0.884881	1.024675
ethnicity_r	0.105065	1.25788	0.953117	1.660092
ethnicity_r	0.265937	0.905671	0.760606	1.078405
ethnicity_r	0.261085	0.846756	0.633529	1.131749
ethnicity_r	0.677406	1.022098	0.92208	1.132966
ethnicity_r	0.783366	0.927939	0.544463	1.581505
ethnicity_r	1.54E-07	0.646624	0.549458	0.760973
ethnicity_r	2.24E-08	0.747779	0.675353	0.827972
ethnicity_r	0.142317	0.650774	0.366605	1.155212
ethnicity_r	0.77963	1.046768	0.759972	1.441795
ethnicity_r	0.403382	0.764617	0.407425	1.434964
ethnicity_r	0.019821	1.244522	1.035325	1.495989
ethnicity_r	0.226437	0.970834	0.92537	1.018533
ethnicity_r	0.171008	1.107181	0.956995	1.280937

ethnicity_r	0	0.770431	0.735633	0.806875
ethnicity_r	0	0.78236	0.744367	0.822292
ethnicity_r	0.501413	1.040295	0.927122	1.167283
ethnicity_r	0.224894	1.132258	0.926452	1.383782
ethnicity_r	1.60E-09	1.261413	1.169763	1.360242
ethnicity_r	0.113274	1.240158	0.950135	1.618709
ethnicity_r	0.476641	1.019656	0.966436	1.075807
ethnicity_r	0.720893	1.035225	0.856156	1.251747
ethnicity_r	0.501336	0.989249	0.958566	1.020914
ethnicity_r	0.000359	0.888709	0.832944	0.948207
ethnicity_r	0.198316	1.143931	0.932003	1.404049
ethnicity_r	0.371218	1.125781	0.868303	1.459608
ethnicity_r	0.045373	1.202922	1.003809	1.441531
ethnicity_r	0.315173	0.910134	0.757366	1.093718
ethnicity_r	0.009828	0.925812	0.87319	0.981606
ethnicity_r	6.78E-05	1.158598	1.07765	1.245626
ethnicity_r	0	0.676144	0.628393	0.727524
ethnicity_r	0.007212	0.925625	0.874884	0.979309
ethnicity_r	0.010528	0.490929	0.284622	0.846775
ethnicity_r	0.264593	0.892038	0.729813	1.090323
ethnicity_r	0.037992	1.23316	1.011677	1.503131
ethnicity_r	1.34E-07	3.453598	2.178712	5.474493
ethnicity_r	0.165776	1.100902	0.960961	1.261223
ethnicity_r	0.049116	1.600783	1.001816	2.557862
ethnicity_r	0.305516	0.957633	0.88153	1.040307
ethnicity_r	5.26E-05	1.540606	1.249445	1.899616
ethnicity_r	0.028718	2.451814	1.097695	5.476378
ethnicity_r	0.003637	2.088452	1.271348	3.430715
ethnicity_r	1.01E-07	1.401745	1.237879	1.587303
ethnicity_r	0.02473	2.176363	1.103957	4.290525
ethnicity_r	0.039819	0.940875	0.88776	0.997169
ethnicity_r	0.847506	1.021857	0.81975	1.273794
ethnicity_r	0.001235	0.922123	0.877863	0.968615
ethnicity_r	0.000139	0.708914	0.593925	0.846166
ethnicity_r	0.175979	0.575269	0.25827	1.28135
ethnicity_r	0.037706	1.493904	1.023061	2.181443
ethnicity_r	0.721264	0.922559	0.592489	1.436507
ethnicity_r	0.628229	0.753426	0.239516	2.369997
ethnicity_r	0.522118	1.021975	0.956166	1.092313
ethnicity_r	0.358667	1.180509	0.828286	1.682511
ethnicity_r	0.27035	0.949397	0.865658	1.041236
ethnicity_r	0	0.738415	0.699545	0.779445
ethnicity_r	0.798011	0.922163	0.495776	1.715262
ethnicity_r	0.111968	1.420262	0.921454	2.18909
ethnicity_r	0.674841	1.124253	0.650464	1.943145
ethnicity_r	0.484596	1.204538	0.714787	2.029855
ethnicity_r	0.06926	1.276781	0.980914	1.661887
ethnicity_r	0.566673	0.662256	0.161727	2.711867
ethnicity_r	0.106534	0.766786	0.555422	1.058584
ethnicity_r	0.472691	0.909392	0.701724	1.178517

ethnicity_r	0.472293	1.293589	0.641112	2.61011
ethnicity_r	0.391486	1.168495	0.818366	1.668423
ethnicity_r	0.869672	0.942766	0.46627	1.906207
ethnicity_e	0.086495	1.043577	0.993902	1.095734
ethnicity_e	5.07E-08	1.275475	1.168587	1.392139
ethnicity_e	0.065917	0.954385	0.908056	1.003077
ethnicity_e	0.056546	1.0885	0.997637	1.187638
ethnicity_e	2.08E-06	1.128907	1.073762	1.186885
ethnicity_e	2.33E-06	1.23952	1.133827	1.355065
ethnicity_e	0.484375	1.024038	0.958079	1.094538
ethnicity_e	0.002938	1.204079	1.065372	1.360844
ethnicity_e	0.000474	1.09249	1.039615	1.148054
ethnicity_e	4.00E-06	1.235599	1.129339	1.351858
ethnicity_e	3.83E-05	0.832051	0.762322	0.90816
ethnicity_e	0.566918	1.036579	0.916631	1.172223
ethnicity_e	0.852067	1.01716	0.850599	1.216336
ethnicity_e	0.217669	1.23648	0.882297	1.732843
ethnicity_e	0.333584	0.973864	0.922962	1.027573
ethnicity_e	0.178481	0.929831	0.836327	1.033789
ethnicity_e	0.201797	0.965491	0.914807	1.018984
ethnicity_e	0.00912	1.126927	1.030118	1.232834
