# Supplementary material for: Inequalities in SARS-CoV-2 case rates by ethnicity, religion, measures of socioeconomic position, English proficiency, and self-reported disability: cohort study of 39 million people in England during the alpha and delta waves
Source: BMJ Med. 2023 Apr 3;2(1):e000187. doi: 10.1136/bmjmed-2022-000187 (PMC10568121; doi:10.1136/bmjmed-2022-000187)
Supplement: Supplementary data [file bmjmed-2022-000187supp003.pdf]

term	p.value	rr	lower_ci	upper_ci
(Intercept)	0	2.90E-09	7.68E-11	1.09E-07
age_1_1	7.20E-12	11.90247	5.86177	24.16826
age_1_2	5.39E-11	4.347023	2.802245	6.743382
age_1_3	1.93E-10	2.766115	2.022258	3.783587
age_1_4	4.58E-07	1.873146	1.467695	2.390604
age_1_5	3.03E-13	2.112172	1.727659	2.582264
age_1_6	6.15E-12	1.843835	1.548747	2.195146
age_1_7	1.67E-12	1.807762	1.533718	2.130772
age_1_8	1.78E-11	1.93905	1.598629	2.351962
age_1_9	0.07641	1.312311	0.971561	1.772571
age_2_1	2.13E-12	0.846863	0.808491	0.887057
age_2_2	8.27E-12	0.940801	0.924479	0.957412
age_2_3	1.92E-11	0.969643	0.960953	0.978411
age_2_4	0.000185	0.989584	0.984167	0.995031
age_2_5	6.66E-16	0.984576	0.980869	0.988298
age_2_6	1.13E-12	0.989327	0.986407	0.992256
age_2_7	4.03E-12	0.990085	0.987301	0.992877
age_2_8	8.71E-10	0.98842	0.984747	0.992107
age_2_9	0.346866	0.997129	0.991172	1.003121
age_3_1	5.77E-12	1.003562	1.002547	1.004579
age_3_2	3.63E-12	1.000818	1.000587	1.001048
age_3_3	1.46E-12	1.000311	1.000225	1.000398
age_3_4	0.026168	1.000047	1.000006	1.000088
age_3_5	2.22E-16	1.0001	1.000076	1.000124
age_3_6	4.75E-11	1.000058	1.000041	1.000075
age_3_7	6.33E-10	1.000051	1.000035	1.000067
age_3_8	1.18E-08	1.000061	1.00004	1.000082
age_3_9	0.47003	1.000012	0.99998	1.000043
sex_1	0	0.938853	0.935454	0.942265
region_1	0	1.270785	1.260188	1.281471
region_2	0	1.105559	1.098522	1.112641
region_3	0	1.158034	1.150015	1.166109
region_4	0	1.1942	1.187008	1.201435
region_5	0	1.386726	1.374282	1.399283
region_6	0	0.902354	0.895533	0.909227
region_7	0.227176	1.005134	0.996815	1.013524
region_8	0	1.179561	1.168897	1.190322
ruralurban	0	1.088857	1.079073	1.09873
ruralurban	0	1.164008	1.155058	1.173028
ruralurban	0	1.162378	1.154192	1.170622
bmi_categ	0	1.257873	1.241258	1.274711
bmi_categ	0	1.17807	1.162629	1.193716
bmi_categ	0	1.273559	1.256673	1.290671
health_cor	0	1.014768	1.012414	1.017127
learning_c	0	1.589124	1.555621	1.623348
ethnicity_	0	0.926676	0.914434	0.939082
ethnicity_	0.003579	0.826893	0.727625	0.939705
ethnicity_	0	0.784029	0.765289	0.803229
ethnicity_	0.235217	0.988981	0.971051	1.007242

ethnicity_5	0	0.734739	0.706643	0.763951
ethnicity_6	0	0.586629	0.546854	0.629297
ethnicity_7	2.81E-07	0.952425	0.934871	0.970309
ethnicity_8	0	0.687685	0.674432	0.701198
ethnicity_9	0.616538	1.077627	0.804239	1.443949
imd_quint	0.319017	0.997217	0.991765	1.002699
imd_quint	6.96E-05	0.988708	0.983191	0.994256
imd_quint	7.33E-06	0.986998	0.981368	0.992661
imd_quint	0	0.968471	0.962502	0.974476
religion_1	0.441105	0.929164	0.770738	1.120154
religion_2	0	0.883352	0.875835	0.890934
religion_3	0	0.797662	0.772755	0.823373
religion_4	0	0.947075	0.942963	0.951206
religion_5	2.32E-07	0.870327	0.825702	0.917364
religion_6	0.211605	1.017841	0.989988	1.046478
religion_7	0.513422	0.941599	0.786116	1.127835
religion_8	1.71E-13	0.830404	0.79036	0.872476
education_	0	1.063067	1.051603	1.074655
education_	6.60E-10	1.067518	1.045609	1.089887
education_	0	1.163839	1.155994	1.171737
education_	0	1.156164	1.147524	1.16487
education_	0	1.121812	1.113861	1.129819
education_	0	1.098605	1.090629	1.10664
education_	0	1.191724	1.17726	1.206365
tenure_1	0	0.92606	0.920919	0.931231
tenure_2	0	0.914186	0.909461	0.918936
tenure_3	0	0.9252	0.91215	0.938437
tenure_4	0	0.768347	0.753419	0.78357
care_home	0	1.32406	1.277118	1.372726
english_lar	2.98E-08	0.865362	0.822224	0.910763
english_lar	1.20E-07	0.749105	0.673121	0.833667
ethnicity_r	0.469634	0.856115	0.56189	1.304407
ethnicity_r	0.227611	0.978495	0.944541	1.013669
ethnicity_r	0.164071	1.088653	0.965893	1.227016
ethnicity_r	2.55E-08	0.939793	0.91948	0.960555
ethnicity_r	0.009405	0.91664	0.85836	0.978877
ethnicity_r	0.233392	0.956488	0.889	1.029099
ethnicity_r	0.38978	0.876105	0.648097	1.184329
ethnicity_r	0.862937	1.012931	0.875451	1.172
ethnicity_r	0.04706	1.41744	1.004535	2.000065
ethnicity_r	0.681939	0.970594	0.84148	1.119519
ethnicity_r	0.459414	0.752775	0.354742	1.597417
ethnicity_r	0.731275	0.96751	0.801289	1.168212
ethnicity_r	0.781903	0.98063	0.853811	1.126285
ethnicity_r	0.363158	1.309166	0.732537	2.339699
ethnicity_r	0.566633	1.118745	0.762161	1.64216
ethnicity_r	0.617943	1.185141	0.607963	2.310271
ethnicity_r	0.011782	1.281056	1.056471	1.553384
ethnicity_r	6.25E-05	1.11933	1.059226	1.182845
ethnicity_r	0.001984	1.279224	1.094376	1.495293

ethnicity_r	0.728091	0.991483	0.944805	1.040466
ethnicity_r	6.43E-06	1.151215	1.082903	1.223837
ethnicity_r	0.760327	0.977658	0.845545	1.130413
ethnicity_r	0.509761	1.064241	0.884389	1.280667
ethnicity_r	0.09635	1.064582	0.988877	1.146082
ethnicity_r	0.645881	0.930836	0.685645	1.26371
ethnicity_r	0.935265	1.002104	0.952545	1.054242
ethnicity_r	0.478775	0.93955	0.790644	1.1165
ethnicity_r	0.080833	1.025568	0.996908	1.055051
ethnicity_r	0.241824	0.955083	0.884339	1.031487
ethnicity_r	0.653239	1.048582	0.852552	1.289686
ethnicity_r	0.748137	0.959811	0.747217	1.232892
ethnicity_r	0.901381	1.010679	0.854374	1.195579
ethnicity_r	0.015987	1.266263	1.045001	1.534375
ethnicity_r	4.05E-07	1.195404	1.115651	1.280859
ethnicity_r	5.41E-07	1.239978	1.139924	1.348813
ethnicity_r	0.001855	1.122822	1.043828	1.207793
ethnicity_r	3.81E-05	1.163159	1.08244	1.249898
ethnicity_r	0.903399	1.034366	0.599375	1.785046
ethnicity_r	0.017994	1.250307	1.039057	1.504507
ethnicity_r	0.000575	1.469751	1.180442	1.829965
ethnicity_r	0.725087	1.175027	0.478235	2.88705
ethnicity_r	0.520784	1.045779	0.912227	1.198884
ethnicity_r	0.963166	1.013538	0.57277	1.793495
ethnicity_r	0.058026	0.924832	0.853043	1.002663
ethnicity_r	0.000104	1.662164	1.285924	2.148484
ethnicity_r	0.999714	3.34E-11	0	Inf
ethnicity_r	0.467309	1.259354	0.676236	2.345292
ethnicity_r	0.004081	1.19469	1.058107	1.348904
ethnicity_r	0.552021	1.353734	0.498946	3.672935
ethnicity_r	0.045029	1.061786	1.001328	1.125895
ethnicity_r	0.005725	1.314274	1.082674	1.595417
ethnicity_r	0.027233	0.946011	0.900537	0.993782
ethnicity_r	0.599848	0.948486	0.778427	1.155697
ethnicity_r	0.180732	0.549347	0.228506	1.32067
ethnicity_r	0.713929	1.084139	0.70388	1.669825
ethnicity_r	0.764755	0.937716	0.61538	1.428892
ethnicity_r	0.968012	1.029029	0.254112	4.167059
ethnicity_r	0.007862	1.112568	1.028406	1.203617
ethnicity_r	0.50785	1.15224	0.7575	1.752683
ethnicity_r	0.557652	1.031191	0.930569	1.142695
ethnicity_r	7.70E-08	1.199473	1.122488	1.281738
ethnicity_r	0.897136	0.948548	0.425854	2.112797
ethnicity_r	0.755846	1.095171	0.61746	1.942474
ethnicity_r	0.996203	0.99841	0.518391	1.922914
ethnicity_r	0.091018	0.443587	0.172811	1.138643
ethnicity_r	0.028888	0.708114	0.519569	0.965081
ethnicity_r	0.999758	1.76E-11	0	Inf
ethnicity_r	0.059937	0.708713	0.495088	1.014516
ethnicity_r	0.252955	0.840624	0.624213	1.132064

ethnicity_r	0.686112	0.80982	0.29113	2.252628
ethnicity_r	0.919643	1.020156	0.692294	1.503289
ethnicity_r	0.193435	0.541208	0.21454	1.365272
ethnicity_e	0.001349	1.091908	1.034752	1.152221
ethnicity_e	0.045385	1.121209	1.002351	1.254162
ethnicity_e	0.004123	1.090323	1.027769	1.156685
ethnicity_e	0.000122	1.255242	1.117827	1.409548
ethnicity_e	0.070516	1.054081	0.9956	1.115996
ethnicity_e	0.010359	1.165156	1.036645	1.309599
ethnicity_e	0.063351	0.931192	0.863688	1.003973
ethnicity_e	0.924274	1.007995	0.855355	1.187873
ethnicity_e	7.14E-07	1.154058	1.0905	1.221321
ethnicity_e	9.58E-07	1.350667	1.197647	1.523238
ethnicity_e	0.017043	0.897026	0.820413	0.980793
ethnicity_e	0.092257	0.875381	0.749731	1.02209
ethnicity_e	0.258067	0.886249	0.7189	1.092554
ethnicity_e	0.625226	0.875174	0.512555	1.494336
ethnicity_e	0.857761	1.005744	0.944683	1.070751
ethnicity_e	0.35788	1.067558	0.928681	1.227202
ethnicity_e	0.015508	1.085435	1.015713	1.159943
ethnicity_e	0.008229	1.177455	1.043094	1.329123
