# Supplementary material for: Inequalities in SARS-CoV-2 case rates by ethnicity, religion, measures of socioeconomic position, English proficiency, and self-reported disability: cohort study of 39 million people in England during the alpha and delta waves
Source: BMJ Med. 2023 Apr 3;2(1):e000187. doi: 10.1136/bmjmed-2022-000187 (PMC10568121; doi:10.1136/bmjmed-2022-000187)
Supplement: Supplementary data [file bmjmed-2022-000187supp004.pdf]

term	level	estimate	std.error	statistic
(Intercept)		-12.31616	0.399649	-30.81745
age_1_1		0.565978	0.080096	7.066219
age_1_2		0.516851	0.049188	10.50758
age_1_3		0.312907	0.035278	8.869803
age_1_4		0.244443	0.02834	8.625275
age_1_5		0.129831	0.02471	5.25422
age_1_6		0.188637	0.026301	7.172341
age_1_7		0.507724	0.033251	15.26935
age_1_8		-0.056029	0.039964	-1.402006
age_1_9		-0.338157	0.059095	-5.722279
age_2_1		-0.038775	0.005248	-7.388445
age_2_2		-0.022791	0.002012	-11.32605
age_2_3		-0.009121	0.001077	-8.466126
age_2_4		-0.005625	0.000727	-7.735279
age_2_5		-0.000974	0.000578	-1.685432
age_2_6		-0.002615	0.000644	-4.059738
age_2_7		-0.012384	0.000807	-15.34635
age_2_8		0.001765	0.000896	1.970882
age_2_9		0.007955	0.001227	6.483868
age_3_1		0.001021	0.000113	9.047022
age_3_2		0.000317	2.74E-05	11.58251
age_3_3		8.72E-05	1.12E-05	7.779684
age_3_4		4.24E-05	6.39E-06	6.639105
age_3_5		-4.89E-06	4.51E-06	-1.084977
age_3_6		6.15E-06	4.69E-06	1.311341
age_3_7		8.07E-05	5.28E-06	15.30282
age_3_8		-8.23E-06	5.22E-06	-1.578602
age_3_9		-4.22E-05	6.45E-06	-6.538634
sex_1	Male	-0.121921	0.001317	-92.58007
region_1	Yorkshire and The Humber	0.108939	0.002796	38.96568
region_2	West Midlands	0.069149	0.002683	25.77518
region_3	South West	-0.348635	0.003164	-110.1754
region_4	North West	0.186206	0.002526	73.72417
region_5	North East	0.13736	0.00337	40.76037
region_6	London	0.064694	0.002865	22.58089
region_7	East of England	0.069779	0.002678	26.05687
region_8	East Midlands	0.130761	0.002813	46.48772
ruralurban_detailed_1	Town and Fringe	0.140309	0.003613	38.83062
ruralurban_detailed_2	Major or minor conurbation	0.378994	0.0031	122.2543
ruralurban_detailed_3	City and Town	0.257137	0.002887	89.07959
bmi_category_Missing		0.15173	0.006664	22.76731
bmi_category_OVERWEIGHT		0.249216	0.006772	36.79966
bmi_category_IDEAL		0.11213	0.006764	16.57655
bmi_category_OBESE		0.357351	0.006776	52.74134
health_condition		0.073489	0.000845	86.99446
learning_condition_No		0.125686	0.006155	20.42178

ethnicity_1	White other		-0.04973	0.005069	-9.811092
ethnicity_2	Pakistani		0.473209	0.035432	13.35531
ethnicity_3	Other		0.120225	0.007267	16.54332
ethnicity_4	Mixed		0.017615	0.006284	2.803318
ethnicity_5	Indian		0.359178	0.010888	32.98968
ethnicity_6	Chinese		-0.49423	0.027306	-18.09964
ethnicity_7	Black Caribbean		-0.0338	0.006928	-4.878378
ethnicity_8	Black African		-0.002219	0.006115	-0.362949
ethnicity_9	Bangladeshi		0.328227	0.098136	3.344615
imd_quintile_1		4	0.06695	0.002168	30.87867
imd_quintile_2		3	0.094138	0.002169	43.40493
imd_quintile_3		2	0.129681	0.002178	59.54505
imd_quintile_4		1	0.154544	0.00229	67.48769
religion_1	Sikh		0.15521	0.06353	2.443118
religion_2	Religion Not Stated		-0.142564	0.003226	-44.18875
religion_3	Other Religion		-0.351516	0.014365	-24.47062
religion_4	No religion		-0.115506	0.00172	-67.13892
religion_5	Muslim		0.253054	0.014862	17.027
religion_6	Jewish		0.05058	0.010408	4.859861
religion_7	Hindu		-0.156713	0.071449	-2.193342
religion_8	Buddhist		-0.342736	0.022921	-14.95306
education_1	Other		0.002911	0.003642	0.799113
education_2	Not classified		-0.00704	0.006415	-1.097539
education_3	Level 4		-0.179316	0.002507	-71.52863
education_4	Level 3		0.000263	0.002804	0.093924
education_5	Level 2		0.010307	0.002584	3.989523
education_6	Level 1		-0.000706	0.002638	-0.267802
education_7	Apprenticeship		0.053508	0.004542	11.77943
tenure_1	Social rented		-0.061785	0.00192	-32.18265
tenure_2	Private rented		-0.101899	0.001963	-51.90289
tenure_3	Other tenure		-0.072641	0.005175	-14.03648
tenure_4	Not classified		-0.184517	0.006994	-26.38351
care_home_1	Yes		1.458451	0.005841	249.7124
english_language_1	Well or Very well		0.059444	0.016864	3.524979
english_language_2	Not well or Not at all		-0.074098	0.02926	-2.532413
ethnicity_religion_1	White other:Sikh		0.308509	0.110966	2.780223
ethnicity_religion_2	White other:Religion Not Sta		-0.057819	0.013289	-4.350893
ethnicity_religion_3	White other:Other Religion		-0.027189	0.054969	-0.494626
ethnicity_religion_4	White other:No religion		-0.178372	0.009219	-19.34843
ethnicity_religion_5	White other:Muslim		0.042463	0.018435	2.303371
ethnicity_religion_6	White other:Jewish		-0.076846	0.027982	-2.746298
ethnicity_religion_7	White other:Hindu		0.294935	0.104074	2.833891
ethnicity_religion_8	White other:Buddhist		-0.049531	0.066326	-0.74678
ethnicity_religion_9	Pakistani:Sikh		-0.037447	0.100002	-0.374461
ethnicity_religion_10	Pakistani:Religion Not State		0.062365	0.038433	1.622685
ethnicity_religion_11	Pakistani:Other Religion		-0.101502	0.207661	-0.488788
ethnicity_religion_12	Pakistani:No religion		-0.307642	0.059262	-5.191202
ethnicity_religion_13	Pakistani:Muslim		-0.25008	0.038454	-6.503302
ethnicity_religion_14	Pakistani:Jewish		-0.347948	0.195943	-1.775759

ethnicity_religion_15	Pakistani:Hindu	0.064457	0.120901	0.533142
ethnicity_religion_16	Pakistani:Buddhist	-0.351434	0.246167	-1.427623
ethnicity_religion_17	Other:Sikh	0.26012	0.064749	4.01737
ethnicity_religion_18	Other:Religion Not Stated	0.005647	0.016924	0.333697
ethnicity_religion_19	Other:Other Religion	0.10738	0.056641	1.895791
ethnicity_religion_20	Other:No religion	-0.246669	0.017235	-14.31208
ethnicity_religion_21	Other:Muslim	-0.211884	0.017263	-12.27413
ethnicity_religion_22	Other:Jewish	-0.048242	0.044926	-1.073816
ethnicity_religion_23	Other:Hindu	0.209993	0.072449	2.898476
ethnicity_religion_24	Other:Buddhist	0.177107	0.028397	6.236856
ethnicity_religion_25	Mixed:Sikh	0.215302	0.083599	2.57541
ethnicity_religion_26	Mixed:Religion Not Stated	0.020966	0.016626	1.261046
ethnicity_religion_27	Mixed:Other Religion	0.044908	0.067068	0.669587
ethnicity_religion_28	Mixed:No religion	0.006317	0.01	0.631706
ethnicity_religion_29	Mixed:Muslim	-0.087426	0.020985	-4.166027
ethnicity_religion_30	Mixed:Jewish	0.012592	0.076755	0.16406
ethnicity_religion_31	Mixed:Hindu	0.219704	0.086616	2.536513
ethnicity_religion_32	Mixed:Buddhist	0.147187	0.059434	2.476474
ethnicity_religion_33	Indian:Sikh	-0.073756	0.064688	-1.140194
ethnicity_religion_34	Indian:Religion Not Stated	-0.04508	0.020421	-2.207526
ethnicity_religion_35	Indian:Other Religion	0.213003	0.027582	7.722609
ethnicity_religion_36	Indian:No religion	-0.377478	0.027086	-13.93611
ethnicity_religion_37	Indian:Muslim	-0.072978	0.019554	-3.732099
ethnicity_religion_38	Indian:Jewish	-0.447381	0.189565	-2.360037
ethnicity_religion_39	Indian:Hindu	-0.043772	0.07241	-0.604506
ethnicity_religion_40	Indian:Buddhist	0.063467	0.08053	0.788115
ethnicity_religion_41	Chinese:Sikh	1.138807	0.187223	6.082633
ethnicity_religion_42	Chinese:Religion Not Statec	0.065515	0.051228	1.278885
ethnicity_religion_43	Chinese:Other Religion	0.338276	0.194763	1.736855
ethnicity_religion_44	Chinese:No religion	-0.022485	0.03122	-0.720189
ethnicity_religion_45	Chinese:Muslim	0.473587	0.081609	5.803131
ethnicity_religion_46	Chinese:Jewish	0.635673	0.379018	1.677155
ethnicity_religion_47	Chinese:Hindu	0.670459	0.210432	3.186114
ethnicity_religion_48	Chinese:Buddhist	0.349102	0.047454	7.356663
ethnicity_religion_49	Black Caribbean:Sikh	0.674126	0.285104	2.364494
ethnicity_religion_50	Black Caribbean:Religion N	-0.037867	0.022643	-1.672345
ethnicity_religion_51	Black Caribbean:Other Reli	-0.04897	0.092285	-0.530633
ethnicity_religion_52	Black Caribbean:No religion	-0.072305	0.019359	-3.735004
ethnicity_religion_53	Black Caribbean:Muslim	-0.25392	0.063494	-3.999107
ethnicity_religion_54	Black Caribbean:Jewish	-0.843187	0.378172	-2.229636
ethnicity_religion_55	Black Caribbean:Hindu	0.474025	0.150648	3.146573
ethnicity_religion_56	Black Caribbean:Buddhist	0.041951	0.170724	0.245722
ethnicity_religion_57	Black African:Sikh	0.081192	0.322603	0.251679
ethnicity_religion_58	Black African:Religion Not S	-0.001279	0.023737	-0.053875
ethnicity_religion_59	Black African:Other Religior	-0.015306	0.151535	-0.101004
ethnicity_religion_60	Black African:No religion	-0.054877	0.036228	-1.514775
ethnicity_religion_61	Black African:Muslim	-0.330072	0.018843	-17.5173
ethnicity_religion_62	Black African:Jewish	0.04197	0.223914	0.187439
ethnicity_religion_63	Black African:Hindu	0.349612	0.175432	1.992859

ethnicity_religion_64	Black African:Buddhist	0.183019	0.224843	0.813986
ethnicity_religion_65	Bangladeshi:Sikh	0.263222	0.179369	1.467487
ethnicity_religion_66	Bangladeshi:Religion Not St	0.20453	0.101051	2.024022
ethnicity_religion_67	Bangladeshi:Other Religion	-0.960636	0.714019	-1.345393
ethnicity_religion_68	Bangladeshi:No religion	-0.210599	0.123082	-1.711053
ethnicity_religion_69	Bangladeshi:Muslim	-0.087278	0.099316	-0.878785
ethnicity_religion_70	Bangladeshi:Jewish	0.205392	0.268747	0.764255
ethnicity_religion_71	Bangladeshi:Hindu	0.202493	0.135703	1.49218
ethnicity_religion_72	Bangladeshi:Buddhist	-0.348157	0.295076	-1.179887
ethnicity_english_1	White other:Well or Very we	0.018612	0.018001	1.033947
ethnicity_english_2	White other:Not well or Not	0.171795	0.030933	5.553804
ethnicity_english_3	Pakistani:Well or Very well	-0.012357	0.018248	-0.677164
ethnicity_english_4	Pakistani:Not well or Not at	0.107679	0.031009	3.472517
ethnicity_english_5	Other:Well or Very well	0.124462	0.018356	6.780535
ethnicity_english_6	Other:Not well or Not at all	0.192631	0.031767	6.063791
ethnicity_english_7	Mixed:Well or Very well	-0.00235	0.02424	-0.096929
ethnicity_english_8	Mixed:Not well or Not at all	0.137359	0.044878	3.060736
ethnicity_english_9	Indian:Well or Very well	0.080342	0.018263	4.399305
ethnicity_english_10	Indian:Not well or Not at all	0.190014	0.032044	5.929738
ethnicity_english_11	Chinese:Well or Very well	-0.281015	0.033017	-8.511317
ethnicity_english_12	Chinese:Not well or Not at a	-0.03968	0.046209	-0.858714
ethnicity_english_13	Black Caribbean:Well or Ve	-0.071763	0.074533	-0.962828
ethnicity_english_14	Black Caribbean:Not well or	0.145778	0.140596	1.036856
ethnicity_english_15	Black African:Well or Very w	0.058566	0.01997	2.932643
ethnicity_english_16	Black African:Not well or No	-0.026838	0.039514	-0.679192
ethnicity_english_17	Bangladeshi:Well or Very w	-0.005305	0.019892	-0.266674
ethnicity_english_18	Bangladeshi:Not well or Not	0.139027	0.032481	4.280264

p.value	Lower CI	Upper CI	Rate ratio	Rate ratio, lower CI	Rate ratio, upper CI
0	-13.09947	-11.53285	4.48E-06	2.05E-06	9.80E-06
1.59E-12	0.408989	0.722966	1.761169	1.505295	2.060536
0	0.420442	0.61326	1.676739	1.522634	1.846441
0	0.243763	0.382051	1.367394	1.276041	1.465287
0	0.188896	0.29999	1.27691	1.207915	1.349845
1.49E-07	0.0814	0.178263	1.138636	1.084805	1.195139
7.37E-13	0.137088	0.240187	1.207603	1.146929	1.271487
0	0.442552	0.572896	1.661505	1.556674	1.773396
0.160914	-0.134358	0.0223	0.945511	0.874277	1.02255
1.05E-08	-0.453983	-0.222331	0.713083	0.635094	0.80065
1.49E-13	-0.049062	-0.028489	0.961967	0.952122	0.971913
0	-0.026735	-0.018847	0.977467	0.973619	0.98133
0	-0.011232	-0.007009	0.990921	0.988831	0.993015
1.02E-14	-0.00705	-0.0042	0.994391	0.992974	0.995809
0.091905	-0.002107	0.000159	0.999026	0.997895	1.000159
4.91E-05	-0.003877	-0.001352	0.997388	0.99613	0.998648
0	-0.013966	-0.010802	0.987692	0.986131	0.989256
0.048737	9.75E-06	0.003521	1.001767	1.00001	1.003527
8.94E-11	0.00555	0.01036	1.007987	1.005566	1.010414
0	0.0008	0.001242	1.001021	1.0008	1.001243
0	0.000263	0.000371	1.000317	1.000263	1.000371
7.11E-15	6.52E-05	0.000109	1.000087	1.000065	1.000109
3.16E-11	2.99E-05	5.49E-05	1.000042	1.00003	1.000055
0.277932	-1.37E-05	3.94E-06	0.999995	0.999986	1.000004
0.189743	-3.04E-06	1.53E-05	1.000006	0.999997	1.000015
0	7.04E-05	9.11E-05	1.000081	1.00007	1.000091
0.114427	-1.85E-05	1.99E-06	0.999992	0.999982	1.000002
6.21E-11	-5.49E-05	-2.96E-05	0.999958	0.999945	0.99997
0	-0.124503	-0.11934	0.885218	0.882936	0.887506
0	0.10346	0.114419	1.115095	1.109001	1.121222
0	0.063891	0.074407	1.071596	1.065976	1.077245
0	-0.354837	-0.342433	0.705651	0.701288	0.710041
0	0.181256	0.191157	1.204671	1.198722	1.210649
0	0.130755	0.143965	1.147241	1.139688	1.154844
0	0.059079	0.070309	1.066833	1.060859	1.07284
0	0.064531	0.075028	1.072272	1.066658	1.077915
0	0.125248	0.136274	1.139696	1.13343	1.145996
0	0.133227	0.147391	1.150629	1.142509	1.158807
0	0.372918	0.38507	1.460814	1.451965	1.469717
0	0.25148	0.262795	1.293223	1.285927	1.30056
0	0.138668	0.164793	1.163846	1.148743	1.179149
0	0.235943	0.26249	1.283019	1.266102	1.300163
0	0.098872	0.125389	1.118659	1.103925	1.133589
0	0.344071	0.370631	1.429537	1.410678	1.448648
0	0.071834	0.075145	1.076257	1.074477	1.078041
0	0.113623	0.137749	1.133926	1.12033	1.147687

0	-0.059665	-0.039796	0.951486	0.94208	0.960986
0	0.403762	0.542656	1.605137	1.497447	1.720571
0	0.105981	0.134469	1.12775	1.111801	1.143929
0.005058	0.005299	0.029932	1.017771	1.005313	1.030384
0	0.337838	0.380518	1.432152	1.401914	1.463042
0	-0.547749	-0.44071	0.610041	0.57825	0.643579
1.07E-06	-0.04738	-0.02022	0.966765	0.953725	0.979983
0.716643	-0.014204	0.009766	0.997783	0.985896	1.009814
0.000824	0.13588	0.520573	1.388504	1.145545	1.682992
0	0.0627	0.071199	1.069242	1.064708	1.073795
0	0.089887	0.098389	1.098711	1.094051	1.103392
0	0.125412	0.13395	1.138465	1.133616	1.143335
0	0.150056	0.159032	1.167125	1.161899	1.172376
0.014561	0.030692	0.279728	1.167903	1.031168	1.32277
0	-0.148887	-0.136241	0.867132	0.861666	0.872633
0	-0.379671	-0.323361	0.703621	0.684086	0.723713
0	-0.118878	-0.112134	0.890915	0.887916	0.893925
0	0.223925	0.282183	1.287953	1.250977	1.326022
1.17E-06	0.030181	0.070979	1.051881	1.030641	1.073559
0.028283	-0.296753	-0.016672	0.85495	0.743227	0.983466
0	-0.38766	-0.297811	0.709826	0.678643	0.742442
0.424225	-0.004228	0.010049	1.002915	0.995781	1.0101
0.272406	-0.019613	0.005532	0.992985	0.980578	1.005548
0	-0.184229	-0.174402	0.835842	0.831745	0.839959
0.925169	-0.005232	0.005759	1.000263	0.994781	1.005776
6.62E-05	0.005243	0.015371	1.010361	1.005257	1.01549
0.788852	-0.005876	0.004463	0.999294	0.994141	1.004473
0	0.044605	0.062411	1.054965	1.045614	1.0644
0	-0.065548	-0.058023	0.940085	0.936554	0.943629
0	-0.105747	-0.098051	0.903121	0.899652	0.906603
0	-0.082784	-0.062498	0.929935	0.92055	0.939415
0	-0.198225	-0.17081	0.831506	0.820185	0.842982
0	1.447003	1.469898	4.299293	4.250358	4.348792
0.000424	0.026391	0.092496	1.061246	1.026742	1.096909
0.011328	-0.131447	-0.016749	0.928581	0.876826	0.983391
0.005432	0.091017	0.526002	1.361394	1.095287	1.692153
1.36E-05	-0.083866	-0.031773	0.943821	0.919555	0.968727
0.620864	-0.134929	0.08055	0.973177	0.873778	1.083883
0	-0.196441	-0.160303	0.836631	0.82165	0.851886
0.021258	0.00633	0.078595	1.043377	1.00635	1.081766
0.006027	-0.131689	-0.022002	0.926033	0.876613	0.978238
0.004598	0.090949	0.49892	1.343039	1.095214	1.646942
0.455196	-0.179529	0.080467	0.951676	0.835664	1.083794
0.708062	-0.233452	0.158558	0.963246	0.791796	1.171819
0.104657	-0.012964	0.137694	1.064351	0.98712	1.147624
0.624992	-0.508517	0.305513	0.903479	0.601387	1.357321
2.09E-07	-0.423795	-0.191488	0.735179	0.654558	0.82573
7.86E-11	-0.325451	-0.17471	0.778738	0.722202	0.839701
0.075773	-0.731997	0.036101	0.706136	0.480948	1.03676

0.593935	-0.172508	0.301423	1.06658	0.841551	1.351781
0.1534	-0.833922	0.131054	0.703678	0.434342	1.140029
5.89E-05	0.133212	0.387028	1.297086	1.142492	1.472597
0.738608	-0.027523	0.038818	1.005663	0.972852	1.039581
0.057988	-0.003637	0.218398	1.113358	0.99637	1.244082
0	-0.28045	-0.212888	0.781399	0.755444	0.808246
0	-0.245718	-0.178049	0.809059	0.782142	0.836901
0.282905	-0.136297	0.039813	0.952903	0.872583	1.040616
0.00375	0.067992	0.351994	1.233669	1.070357	1.4219
4.46E-10	0.121449	0.232765	1.193759	1.129132	1.262085
0.010012	0.051448	0.379155	1.240236	1.052794	1.46105
0.207292	-0.011621	0.053553	1.021187	0.988447	1.055012
0.503121	-0.086545	0.17636	1.045931	0.917094	1.192868
0.527579	-0.013283	0.025917	1.006337	0.986805	1.026255
3.10E-05	-0.128558	-0.046295	0.916287	0.879363	0.954761
0.869684	-0.137847	0.163032	1.012672	0.871232	1.177075
0.011196	0.049936	0.389472	1.245708	1.051203	1.476201
0.013269	0.030696	0.263678	1.158571	1.031172	1.30171
0.254205	-0.200544	0.053031	0.928898	0.818285	1.054463
0.027277	-0.085105	-0.005055	0.955921	0.918416	0.994958
1.15E-14	0.158943	0.267063	1.237388	1.172271	1.306123
0	-0.430568	-0.324389	0.685588	0.65014	0.722969
0.00019	-0.111304	-0.034652	0.929621	0.894667	0.965942
0.018273	-0.818929	-0.075833	0.6393	0.440904	0.926971
0.545507	-0.185695	0.098151	0.957172	0.830527	1.103129
0.43063	-0.094372	0.221306	1.065524	0.909944	1.247705
1.18E-09	0.77185	1.505763	3.123039	2.163766	4.507591
0.200938	-0.034892	0.165923	1.067709	0.965709	1.180482
0.082413	-0.043461	0.720012	1.402527	0.95747	2.054458
0.471409	-0.083677	0.038707	0.977766	0.919729	1.039466
6.51E-09	0.313633	0.63354	1.605743	1.368388	1.884269
0.093512	-0.107203	1.378549	1.888292	0.898343	3.969138
0.001442	0.258013	1.082905	1.955135	1.294356	2.953246
1.89E-13	0.256093	0.442112	1.417794	1.291872	1.555989
0.018055	0.115323	1.232929	1.962317	1.122235	3.431265
0.094456	-0.082246	0.006513	0.962841	0.921045	1.006535
0.595673	-0.229849	0.131909	0.95221	0.794654	1.141005
0.000188	-0.110248	-0.034362	0.930247	0.895612	0.966222
6.36E-05	-0.378368	-0.129471	0.775754	0.684978	0.87856
0.025772	-1.584405	-0.101969	0.430337	0.20507	0.903058
0.001652	0.178755	0.769296	1.606448	1.195728	2.158246
0.805897	-0.292669	0.37657	1.042843	0.746269	1.457278
0.801289	-0.551109	0.713494	1.08458	0.57631	2.04111
0.957035	-0.047803	0.045245	0.998722	0.953322	1.046284
0.919547	-0.312314	0.281703	0.984811	0.731752	1.325384
0.129829	-0.125883	0.016129	0.946602	0.881718	1.01626
0	-0.367003	-0.29314	0.718872	0.692808	0.745918
0.851317	-0.396901	0.480842	1.042863	0.6724	1.617435
0.046277	0.005765	0.69346	1.418517	1.005781	2.000625

0.415653	-0.257673	0.62371	1.200837	0.772848	1.865838
0.142244	-0.088342	0.614787	1.301116	0.915448	1.849262
0.042968	0.00647	0.402591	1.226948	1.00649	1.495695
0.178498	-2.360114	0.438841	0.382649	0.094409	1.550909
0.087071	-0.45184	0.030641	0.810099	0.636456	1.031115
0.379518	-0.281938	0.107382	0.916423	0.754321	1.11336
0.444715	-0.321353	0.732136	1.228006	0.725167	2.079519
0.135652	-0.063485	0.468472	1.224452	0.938489	1.597551
0.238045	-0.926506	0.230193	0.705988	0.395935	1.258843
0.301161	-0.01667	0.053894	1.018786	0.983468	1.055372
2.80E-08	0.111167	0.232423	1.187434	1.117581	1.261654
0.498302	-0.048124	0.02341	0.987719	0.953016	1.023686
0.000516	0.046902	0.168457	1.113691	1.048019	1.183477
1.20E-11	0.088485	0.160439	1.132539	1.092518	1.174027
1.33E-09	0.130367	0.254895	1.212435	1.139246	1.290326
0.922782	-0.04986	0.045161	0.997653	0.951362	1.046196
0.002208	0.049399	0.225319	1.14724	1.050639	1.252723
1.09E-05	0.044548	0.116137	1.083658	1.045555	1.12315
3.03E-09	0.127207	0.252821	1.209266	1.135652	1.287652
0	-0.345728	-0.216302	0.755017	0.707705	0.805492
0.390498	-0.13025	0.050889	0.961096	0.877876	1.052207
0.335634	-0.217848	0.074323	0.930752	0.804247	1.077154
0.299803	-0.12979	0.421346	1.156939	0.878279	1.524012
0.003361	0.019424	0.097708	1.060315	1.019614	1.10264
0.497016	-0.104286	0.05061	0.973519	0.900968	1.051913
0.78972	-0.044292	0.033683	0.994709	0.956674	1.034257
1.87E-05	0.075364	0.20269	1.149155	1.078277	1.224692
