# Supplementary material for: Inequalities in SARS-CoV-2 case rates by ethnicity, religion, measures of socioeconomic position, English proficiency, and self-reported disability: cohort study of 39 million people in England during the alpha and delta waves
Source: BMJ Med. 2023 Apr 3;2(1):e000187. doi: 10.1136/bmjmed-2022-000187 (PMC10568121; doi:10.1136/bmjmed-2022-000187)
Supplement: Supplementary data [file bmjmed-2022-000187supp005.pdf]

term	level	estimate	std.error	statistic	p.value	Lower CI
(Intercept)		-17.27719	0.317296	-54.45136	0	-17.89909
age_1_1		1.938695	0.06309	30.72914	0	1.815039
age_1_2		1.190198	0.038853	30.6331	0	1.114046
age_1_3		0.836834	0.028147	29.7308	0	0.781666
age_1_4		0.485842	0.022655	21.44508	0	0.441438
age_1_5		0.625906	0.020554	30.45235	0	0.585621
age_1_6		0.517778	0.023406	22.12139	0	0.471902
age_1_7		0.487616	0.033185	14.69399	0	0.422574
age_1_8		0.53903	0.059243	9.098621	0	0.422913
age_1_9		0.247599	0.107	2.314001	0.020668	0.037878
age_2_1		-0.120091	0.00412	-29.15101	0	-0.128166
age_2_2		-0.049387	0.00159	-31.05886	0	-0.052504
age_2_3		-0.025916	0.000871	-29.76313	0	-0.027623
age_2_4		-0.007418	0.000589	-12.59559	0	-0.008573
age_2_5		-0.013269	0.00051	-25.99347	0	-0.01427
age_2_6		-0.009379	0.000607	-15.46068	0	-0.010568
age_2_7		-0.008343	0.000838	-9.957565	0	-0.009985
age_2_8		-0.009678	0.00138	-7.0151	2.30E-12	-0.012382
age_2_9		-0.00311	0.002251	-1.381712	0.16706	-0.007521
age_3_1		0.002311	8.85E-05	26.10778	0	0.002138
age_3_2		0.000658	2.18E-05	30.19865	0	0.000615
age_3_3		0.000267	9.20E-06	29.03021	0	0.000249
age_3_4		2.50E-05	5.23E-06	4.782926	1.73E-06	1.48E-05
age_3_5		8.61E-05	4.12E-06	20.90914	0	7.80E-05
age_3_6		5.12E-05	4.49E-06	11.40813	0	4.24E-05
age_3_7		4.26E-05	5.54E-06	7.684878	1.53E-14	3.17E-05
age_3_8		5.12E-05	8.11E-06	6.304387	2.89E-10	3.53E-05
age_3_9		1.41E-05	1.19E-05	1.189298	0.234322	-9.14E-06
sex_1	Male	-0.079654	0.001114	-71.51281	0	-0.081837
region_1	Yorkshire and T	0.195598	0.002281	85.76864	0	0.191128
region_2	West Midlands	0.080169	0.002262	35.4476	0	0.075736
region_3	South West	0.140485	0.00218	64.42914	0	0.136211
region_4	North West	0.132454	0.002137	61.96738	0	0.128264
region_5	North East	0.270278	0.002716	99.51271	0	0.264955
region_6	London	-0.130113	0.002597	-50.09148	0	-0.135204
region_7	East of Englanc	-0.008564	0.002212	-3.870894	0.000108	-0.012901
region_8	East Midlands	0.149781	0.002297	65.21755	0	0.14528
ruralurban_	Town and Fring	0.076103	0.002665	28.55432	0	0.070879
ruralurban_	Major or minor c	0.102496	0.002383	43.00473	0	0.097825
ruralurban_	City and Town	0.114246	0.002132	53.57909	0	0.110067
bmi_category_Missing		0.144449	0.006619	21.82315	0	0.131476
bmi_category_OVERWEIG		0.227598	0.00674	33.77054	0	0.214388
bmi_category_IDEAL		0.158105	0.006711	23.55949	0	0.144952
bmi_category_OBESE		0.240234	0.00676	35.53506	0	0.226984
health_condition		0.015614	0.000901	17.32053	0	0.013847
learning_condition_No		0.388887	0.005853	66.44492	0	0.377415

ethnicity_1	White other	-0.122717	0.004561	-26.90724	0	-0.131656
ethnicity_2	Pakistani	-0.341839	0.045398	-7.52981	5.08E-14	-0.430819
ethnicity_3	Other	-0.304787	0.007665	-39.7634	0	-0.31981
ethnicity_4	Mixed	-0.063547	0.005076	-12.51937	0	-0.073496
ethnicity_5	Indian	-0.34792	0.012199	-28.52043	0	-0.37183
ethnicity_6	Chinese	-0.570873	0.023609	-24.18037	0	-0.617146
ethnicity_7	Black Caribbeai	-0.099737	0.006828	-14.60719	0	-0.113119
ethnicity_8	Black African	-0.437605	0.00627	-69.79885	0	-0.449894
ethnicity_9	Bangladeshi	-0.104	0.106101	-0.980199	0.326988	-0.311958
imd_quintile	4	-0.011613	0.001705	-6.810477	9.73E-12	-0.014955
imd_quintile	3	-0.022381	0.00174	-12.8639	0	-0.025792
imd_quintile	2	-0.031468	0.001793	-17.54762	0	-0.034983
imd_quintile	1	-0.066194	0.001931	-34.27328	0	-0.06998
religion_1	Sikh	-0.13295	0.059674	-2.227923	0.025886	-0.249912
religion_2	Religion Not Sta	-0.103074	0.00256	-40.26917	0	-0.108091
religion_3	Other Religion	-0.237833	0.011588	-20.52374	0	-0.260545
religion_4	No religion	-0.045231	0.001331	-33.97574	0	-0.047841
religion_5	Muslim	-0.251791	0.015463	-16.28383	0	-0.282097
religion_6	Jewish	-0.066793	0.009642	-6.927182	4.29E-12	-0.085692
religion_7	Hindu	-0.100204	0.055919	-1.791944	0.073142	-0.209806
religion_8	Buddhist	-0.203831	0.018085	-11.27093	0	-0.239277
education_	Other	0.063637	0.004121	15.44286	0	0.055561
education_	Not classified	0.061561	0.006043	10.18759	0	0.049717
education_	Level 4	0.129846	0.002514	51.65439	0	0.124919
education_	Level 3	0.123322	0.002781	44.34313	0	0.117871
education_	Level 2	0.099729	0.002634	37.86059	0	0.094566
education_	Level 1	0.085399	0.00271	31.51755	0	0.080089
education_	Apprenticeship	0.152258	0.004455	34.17774	0	0.143526
tenure_1	Social rented	-0.096497	0.001708	-56.48038	0	-0.099845
tenure_2	Private rented	-0.100696	0.001603	-62.82646	0	-0.103837
tenure_3	Other tenure	-0.076889	0.004495	-17.10369	0	-0.0857
tenure_4	Not classified	-0.284669	0.007196	-39.5592	0	-0.298774
care_home	Yes	0.27754	0.013721	20.22801	0	0.250648
english_lan	Well or Very we	-0.169181	0.016632	-10.17191	0	-0.20178
english_lan	Not well or Not a	-0.391645	0.028273	-13.85214	0	-0.447061
ethnicity_re	White other:Sikl	-0.250638	0.143255	-1.749598	0.080188	-0.531417
ethnicity_re	White other:Rel	-0.039496	0.011487	-3.438338	0.000585	-0.06201
ethnicity_re	White other:Oth	0.039176	0.045656	0.858065	0.390856	-0.05031
ethnicity_re	White other:No	-0.079403	0.007588	-10.46395	0	-0.094276
ethnicity_re	White other:Mu:	0.036605	0.020613	1.775811	0.075764	-0.003797
ethnicity_re	White other:Jew	-0.035925	0.026481	-1.356649	0.174893	-0.087827
ethnicity_re	White other:Hin	-0.155274	0.102956	-1.508161	0.131513	-0.357068
ethnicity_re	White other:Buc	0.041184	0.054558	0.754868	0.450328	-0.06575
ethnicity_re	Pakistani:Sikh	0.399728	0.111005	3.600991	0.000317	0.182158
ethnicity_re	Pakistani:Religi	-0.098911	0.048984	-2.019255	0.043461	-0.19492
ethnicity_re	Pakistani:Other	-0.150103	0.262402	-0.572033	0.5673	-0.664411
ethnicity_re	Pakistani:No rel	0.029036	0.066335	0.437709	0.661597	-0.100982
ethnicity_re	Pakistani:Muslir	0.086795	0.048032	1.807002	0.070762	-0.007349
ethnicity_re	Pakistani:Jewisl	-0.106893	0.228351	-0.468111	0.639705	-0.554461

ethnicity_re	Pakistani:Hindu	-0.104523	0.141007	-0.741261	0.458535	-0.380897
ethnicity_re	Pakistani:Buddh	0.450729	0.209874	2.14762	0.031744	0.039377
ethnicity_re	Other:Sikh	0.279661	0.061537	4.544601	5.50E-06	0.159048
ethnicity_re	Other:Religion N	0.036557	0.017455	2.094325	0.036231	0.002345
ethnicity_re	Other:Other Rel	0.378322	0.053473	7.075021	1.49E-12	0.273515
ethnicity_re	Other:No religio	0.006743	0.016382	0.411647	0.680598	-0.025365
ethnicity_re	Other:Muslim	0.18432	0.018353	10.04289	0	0.148347
ethnicity_re	Other:Jewish	-0.017052	0.050952	-0.334665	0.737878	-0.116918
ethnicity_re	Other:Hindu	0.098648	0.057584	1.713133	0.086688	-0.014216
ethnicity_re	Other:Buddhist	0.086501	0.025996	3.32751	0.000876	0.035549
ethnicity_re	Mixed:Sikh	0.015176	0.078503	0.193311	0.846715	-0.13869
ethnicity_re	Mixed:Religion I	0.003173	0.012578	0.252258	0.800841	-0.021481
ethnicity_re	Mixed:Other Re	0.005701	0.053626	0.106305	0.91534	-0.099406
ethnicity_re	Mixed:No religic	0.034964	0.007641	4.575887	4.74E-06	0.019988
ethnicity_re	Mixed:Muslim	0.059664	0.020644	2.890096	0.003851	0.019201
ethnicity_re	Mixed:Jewish	0.03619	0.068738	0.526488	0.598549	-0.098536
ethnicity_re	Mixed:Hindu	-0.021497	0.068878	-0.3121	0.754965	-0.156497
ethnicity_re	Mixed:Buddhist	0.017037	0.045832	0.371717	0.710103	-0.072795
ethnicity_re	Indian:Sikh	0.271964	0.061271	4.438678	9.05E-06	0.151872
ethnicity_re	Indian:Religion	0.13379	0.021554	6.207101	5.40E-10	0.091543
ethnicity_re	Indian:Other Re	0.317661	0.029331	10.83012	0	0.260172
ethnicity_re	Indian:No religic	0.10124	0.025463	3.975943	7.01E-05	0.051332
ethnicity_re	Indian:Muslim	0.153486	0.021818	7.034781	2.00E-12	0.110722
ethnicity_re	Indian:Jewish	-0.148304	0.229928	-0.645002	0.518926	-0.598962
ethnicity_re	Indian:Hindu	0.26343	0.057408	4.588704	4.46E-06	0.15091
ethnicity_re	Indian:Buddhist	0.346425	0.07991	4.335189	1.46E-05	0.189802
ethnicity_re	Chinese:Sikh	0.225255	0.308234	0.730793	0.464906	-0.378884
ethnicity_re	Chinese:Religio	0.026058	0.044057	0.591465	0.554209	-0.060293
ethnicity_re	Chinese:Other I	0.507236	0.158268	3.204924	0.001351	0.197031
ethnicity_re	Chinese:No reli	-0.049386	0.026955	-1.83216	0.066928	-0.102217
ethnicity_re	Chinese:Muslim	0.561463	0.089333	6.285021	3.28E-10	0.386369
ethnicity_re	Chinese:Jewish	-24.0688	51867.12	-0.000464	0.99963	-101683.6
ethnicity_re	Chinese:Hindu	0.328581	0.212938	1.543078	0.122812	-0.088779
ethnicity_re	Chinese:Buddhi	0.200278	0.0417	4.802859	1.56E-06	0.118547
ethnicity_re	Black Caribbea	0.162612	0.412845	0.393881	0.693669	-0.646565
ethnicity_re	Black Caribbea	-0.019245	0.021382	-0.90007	0.368083	-0.061154
ethnicity_re	Black Caribbea	0.187777	0.078304	2.398066	0.016482	0.034302
ethnicity_re	Black Caribbea	-0.062543	0.01812	-3.45163	0.000557	-0.098057
ethnicity_re	Black Caribbea	0.074215	0.063955	1.16042	0.245878	-0.051137
ethnicity_re	Black Caribbea	-0.260117	0.316447	-0.821991	0.411082	-0.880352
ethnicity_re	Black Caribbea	0.057597	0.167845	0.343157	0.731481	-0.271379
ethnicity_re	Black Caribbea	0.035987	0.16338	0.220263	0.825667	-0.284239
ethnicity_re	Black African:Si	0.205516	0.382701	0.537015	0.591257	-0.544578
ethnicity_re	Black African:R	-0.013309	0.023588	-0.564243	0.572589	-0.059542
ethnicity_re	Black African:O	-0.065355	0.167169	-0.390948	0.695835	-0.393007
ethnicity_re	Black African:N	-0.021895	0.037655	-0.58147	0.560924	-0.0957
ethnicity_re	Black African:M	0.181888	0.019848	9.164131	0	0.142986
ethnicity_re	Black African:Je	-0.491482	0.353732	-1.38942	0.164705	-1.184795
ethnicity_re	Black African:Hi	0.214691	0.200497	1.070793	0.284262	-0.178283

ethnicity_re	Black African:Br	0.254012	0.236465	1.074208	0.282729	-0.209458
ethnicity_re	Bangladeshi:Sik	-0.375304	0.259677	-1.44527	0.148382	-0.884272
ethnicity_re	Bangladeshi:Re	-0.238775	0.11029	-2.164972	0.03039	-0.454944
ethnicity_re	Bangladeshi:Ot	-24.66163	57876.63	-0.000426	0.99966	-113462.9
ethnicity_re	Bangladeshi:No	-0.110557	0.127466	-0.867339	0.385756	-0.360391
ethnicity_re	Bangladeshi:Mu	-0.051668	0.107346	-0.481321	0.630288	-0.262067
ethnicity_re	Bangladeshi:Je	-0.521619	0.421899	-1.236361	0.216324	-1.348541
ethnicity_re	Bangladeshi:Hin	-0.062006	0.140014	-0.442856	0.65787	-0.336433
ethnicity_re	Bangladeshi:Bu	-0.434715	0.308069	-1.411095	0.158217	-1.038531
ethnicity_en	White other:We	0.101328	0.017597	5.758244	8.50E-09	0.066838
ethnicity_en	White other:Not	0.162234	0.029814	5.441595	5.28E-08	0.103799
ethnicity_en	Pakistani:Well c	0.181734	0.019293	9.419728	0	0.14392
ethnicity_en	Pakistani:Not w	0.336333	0.032327	10.40397	0	0.272971
ethnicity_en	Other:Well or V	0.103133	0.018611	5.541411	3.00E-08	0.066655
ethnicity_en	Other:Not well c	0.23011	0.032139	7.159948	8.07E-13	0.167119
ethnicity_en	Mixed:Well or V	-0.065418	0.024676	-2.651042	0.008024	-0.113784
ethnicity_en	Mixed:Not well c	0.025135	0.046598	0.539396	0.589613	-0.066197
ethnicity_en	Indian:Well or V	0.144229	0.018646	7.735008	1.02E-14	0.107682
ethnicity_en	Indian:Not well c	0.324111	0.033334	9.72303	0	0.258776
ethnicity_en	Chinese:Well or	-0.153156	0.030279	-5.058153	4.23E-07	-0.212502
ethnicity_en	Chinese:Not we	0.081837	0.045057	1.816292	0.069326	-0.006475
ethnicity_en	Black Caribbean	-0.050796	0.077917	-0.65192	0.514453	-0.203514
ethnicity_en	Black Caribbean	-0.077033	0.194629	-0.395792	0.692259	-0.458506
ethnicity_en	Black African:W	0.14546	0.02079	6.996577	2.62E-12	0.104711
ethnicity_en	Black African:N	0.147166	0.041989	3.504879	0.000457	0.064868
ethnicity_en	Bangladeshi:We	0.153423	0.021963	6.985653	2.84E-12	0.110376
ethnicity_en	Bangladeshi:No	0.299731	0.035391	8.469181	0	0.230365

Upper CI	Rate ratio	Rate ratio, lower CI	Rate ratio, upper CI
-16.65529	3.14E-08	1.68E-08	5.84E-08
2.062351	6.949674	6.141314	7.864435
1.266351	3.287733	3.04666	3.547882
0.892002	2.309045	2.185109	2.44001
0.530246	1.625544	1.554942	1.699351
0.666191	1.869939	1.796105	1.946807
0.563654	1.678295	1.60304	1.757082
0.552659	1.62843	1.525885	1.737867
0.655146	1.714343	1.526402	1.925424
0.457319	1.280946	1.038605	1.579833
-0.112017	0.88684	0.879708	0.894029
-0.04627	0.951813	0.948851	0.954784
-0.02421	0.974417	0.972755	0.976081
-0.006264	0.992609	0.991464	0.993756
-0.012269	0.986818	0.985832	0.987806
-0.00819	0.990665	0.989488	0.991844
-0.006701	0.991692	0.990064	0.993322
-0.006974	0.990368	0.987694	0.99305
0.001302	0.996895	0.992507	1.001302
0.002485	1.002314	1.00214	1.002488
0.000701	1.000658	1.000616	1.000701
0.000285	1.000267	1.000249	1.000285
3.53E-05	1.000025	1.000015	1.000035
9.42E-05	1.000086	1.000078	1.000094
6.00E-05	1.000051	1.000042	1.00006
5.34E-05	1.000043	1.000032	1.000053
6.71E-05	1.000051	1.000035	1.000067
3.73E-05	1.000014	0.999991	1.000037
-0.077471	0.923436	0.921422	0.925454
0.200068	1.216038	1.210615	1.221486
0.084602	1.08347	1.078678	1.088284
0.144759	1.150832	1.145924	1.15576
0.136643	1.141626	1.136853	1.146419
0.275602	1.310329	1.303372	1.317323
-0.125021	0.877997	0.873538	0.882478
-0.004228	0.991472	0.987182	0.995781
0.154283	1.16158	1.156363	1.166821
0.081327	1.079074	1.073451	1.084725
0.107168	1.107933	1.10277	1.113121
0.118426	1.121028	1.116353	1.125723
0.157422	1.155403	1.14051	1.17049
0.240807	1.25558	1.239104	1.272276
0.171259	1.17129	1.155984	1.186798
0.253485	1.271547	1.254809	1.288508
0.01738	1.015736	1.013943	1.017532
0.400358	1.475337	1.45851	1.492359

-0.113778	0.884514	0.876643	0.892456
-0.252859	0.710462	0.649976	0.776577
-0.289764	0.73728	0.726287	0.748441
-0.053598	0.93843	0.92914	0.947813
-0.32401	0.706155	0.689471	0.723243
-0.524599	0.565032	0.539482	0.591792
-0.086354	0.905076	0.893044	0.91727
-0.425317	0.645581	0.637696	0.653563
0.103958	0.901225	0.732013	1.109554
-0.008271	0.988454	0.985156	0.991763
-0.018971	0.977867	0.974538	0.981207
-0.027953	0.969022	0.965622	0.972434
-0.062409	0.935949	0.932413	0.939499
-0.015988	0.875509	0.778869	0.984139
-0.098057	0.902061	0.897546	0.906597
-0.21512	0.788335	0.770631	0.806445
-0.042622	0.955776	0.953286	0.958274
-0.221484	0.777407	0.7542	0.801329
-0.047895	0.935388	0.917877	0.953234
0.009398	0.904653	0.810741	1.009442
-0.168385	0.8156	0.787197	0.845028
0.071714	1.065706	1.057133	1.074348
0.073405	1.063495	1.050974	1.076166
0.134773	1.138653	1.133056	1.144276
0.128773	1.131248	1.125099	1.137431
0.104892	1.104872	1.099182	1.110591
0.09071	1.089152	1.083383	1.094952
0.16099	1.164461	1.154337	1.174673
-0.093148	0.908013	0.904977	0.911059
-0.097554	0.904208	0.901372	0.907053
-0.068078	0.925993	0.91787	0.934188
-0.270565	0.752263	0.741727	0.762948
0.304433	1.319879	1.284858	1.355856
-0.136582	0.844356	0.817275	0.872335
-0.336229	0.675944	0.639505	0.714459
0.030141	0.778304	0.587771	1.0306
-0.016982	0.961274	0.939873	0.983162
0.128662	1.039953	0.950935	1.137306
-0.06453	0.923668	0.910032	0.937508
0.077007	1.037283	0.99621	1.080049
0.015977	0.964713	0.91592	1.016105
0.04652	0.85618	0.699725	1.047619
0.148118	1.042044	0.936365	1.15965
0.617298	1.491419	1.199804	1.853912
-0.002903	0.905823	0.8229	0.997102
0.364206	0.860619	0.514576	1.43937
0.159053	1.029461	0.903949	1.1724
0.180938	1.090673	0.992678	1.198341
0.340674	0.898621	0.574382	1.405895

0.171851	0.900754	0.683248	1.187501
0.862081	1.569456	1.040162	2.368085
0.400273	1.322681	1.172395	1.492232
0.070769	1.037233	1.002347	1.073333
0.483128	1.459832	1.314577	1.621138
0.038852	1.006766	0.974954	1.039616
0.220292	1.2024	1.159916	1.24644
0.082815	0.983093	0.889658	1.08634
0.211512	1.103678	0.985885	1.235545
0.137452	1.090352	1.036189	1.147347
0.169041	1.015291	0.870498	1.184169
0.027827	1.003178	0.978749	1.028217
0.110807	1.005717	0.905375	1.11718
0.04994	1.035582	1.020189	1.051208
0.100126	1.06148	1.019387	1.105311
0.170915	1.036852	0.906163	1.18639
0.113504	0.978733	0.855134	1.120196
0.106868	1.017183	0.929791	1.112788
0.392056	1.31254	1.164011	1.48002
0.176036	1.143152	1.095864	1.192481
0.37515	1.37391	1.297153	1.45521
0.151147	1.106542	1.052672	1.163168
0.196249	1.165891	1.117084	1.21683
0.302354	0.862169	0.549382	1.35304
0.375951	1.301387	1.162892	1.456376
0.503049	1.414004	1.20901	1.653756
0.829394	1.252642	0.684625	2.29193
0.112409	1.0264	0.941489	1.11897
0.817441	1.660695	1.217782	2.264697
0.003446	0.951814	0.902834	1.003452
0.736556	1.753235	1.471628	2.08873
101635.5	3.52E-11	0	Inf
0.74594	1.388995	0.915048	2.108422
0.28201	1.221743	1.125859	1.325792
0.971789	1.17658	0.523842	2.642668
0.022663	0.980939	0.940679	1.022922
0.341252	1.206565	1.034897	1.406708
-0.027028	0.939373	0.906597	0.973334
0.199567	1.077038	0.950148	1.220874
0.360119	0.770962	0.414637	1.4335
0.386574	1.059288	0.762327	1.471929
0.356212	1.036642	0.752587	1.42791
0.95561	1.228159	0.580087	2.600256
0.032923	0.986779	0.942196	1.033471
0.262298	0.936735	0.675024	1.299913
0.051909	0.978343	0.908737	1.05328
0.220789	1.199479	1.153714	1.24706
0.201832	0.611719	0.305809	1.223643
0.607665	1.239479	0.836705	1.836139

0.717483	1.289187	0.811023	2.049268
0.133664	0.68708	0.413015	1.143008
-0.022606	0.787592	0.634483	0.977647
113413.5	1.95E-11	0	Inf
0.139278	0.895336	0.697404	1.149443
0.158731	0.949644	0.76946	1.172022
0.305302	0.593559	0.259619	1.357035
0.212421	0.939877	0.714314	1.236669
0.169101	0.647449	0.353974	1.18424
0.135818	1.10664	1.069122	1.145474
0.220668	1.176135	1.109377	1.24691
0.219548	1.199295	1.154792	1.245514
0.399694	1.399805	1.313862	1.491369
0.139611	1.108639	1.068927	1.149827
0.293102	1.258739	1.181895	1.34058
-0.017052	0.936676	0.892451	0.983092
0.116466	1.025453	0.935946	1.12352
0.180776	1.155149	1.113694	1.198147
0.389446	1.382801	1.295343	1.476163
-0.093809	0.857996	0.808558	0.910457
0.170149	1.085279	0.993546	1.185481
0.101922	0.950473	0.815859	1.107297
0.304441	0.92586	0.632228	1.355866
0.186209	1.156572	1.11039	1.204674
0.229464	1.158546	1.067018	1.257925
0.19647	1.165818	1.116698	1.217099
0.369097	1.349496	1.25906	1.446428
